# Real-world effectiveness and factors associated with effectiveness of inactivated SARS-CoV-2 vaccines: a systematic review and meta-regression analysis

**DOI:** 10.1186/s12916-023-02861-3

**Published:** 2023-04-27

**Authors:** Shiyao Xu, Jincheng Li, Hongyuan Wang, Fuzhen Wang, Zundong Yin, Zhifeng Wang

**Affiliations:** 1 Department of Health Policy and Management, School of Public Health, Peking University, Beijing, China; 2 Department of Epidemiology and Biostatistics, School of Public Health, Peking University, Beijing, China; 3 Chinese Center for Disease Control and Prevention, National Immunization Programme, Beijing, China

**Keywords:** SARS-CoV-2, Inactivated vaccine, Effectiveness, Factor, Meta-regression

## Abstract

**Background:**

The two inactivated SARS-CoV-2 vaccines, CoronaVac and BBIBP-CorV, have been widely used to control the COVID-19 pandemic. The influence of multiple factors on inactivated vaccine effectiveness (VE) during long-term use and against variants is not well understood.

**Methods:**

We selected published or preprinted articles from PubMed, Embase, Scopus, Web of Science, medRxiv, BioRxiv, and the WHO COVID-19 database by 31 August 2022. We included observational studies that assessed the VE of completed primary series or homologous booster against SARS-CoV-2 infection or severe COVID-19. We used DerSimonian and Laird random-effects models to calculate pooled estimates and conducted multiple meta-regression with an information theoretic approach based on Akaike’s Information Criterion to select the model and identify the factors associated with VE.

**Results:**

Fifty-one eligible studies with 151 estimates were included. For prevention of infection, VE associated with study region, variants, and time since vaccination; VE was significantly decreased against Omicron compared to Alpha (*P* = 0.021), primary series VE was 52.8% (95% CI, 43.3 to 60.7%) against Delta and 16.4% (95% CI, 9.5 to 22.8%) against Omicron, and booster dose VE was 65.2% (95% CI, 48.3 to 76.6%) against Delta and 20.3% (95% CI, 10.5 to 28.0%) against Omicron; primary VE decreased significantly after 180 days (*P* = 0.022). For the prevention of severe COVID-19, VE associated with vaccine doses, age, study region, variants, study design, and study population type; booster VE increased significantly (*P* = 0.001) compared to primary; though VE decreased significantly against Gamma (*P* = 0.034), Delta (*P* = 0.001), and Omicron (*P* = 0.001) compared to Alpha, primary and booster VEs were all above 60% against each variant.

**Conclusions:**

Inactivated vaccine protection against SARS-CoV-2 infection was moderate, decreased significantly after 6 months following primary vaccination, and was restored by booster vaccination. VE against severe COVID-19 was greatest after boosting and did not decrease over time, sustained for over 6 months after the primary series, and more evidence is needed to assess the duration of booster VE. VE varied by variants, most notably against Omicron. It is necessary to ensure booster vaccination of everyone eligible for SARS-CoV-2 vaccines and continue monitoring virus evolution and VE.

**Trial registration:**

PROSPERO, CRD42022353272.

**Supplementary Information:**

The online version contains supplementary material available at 10.1186/s12916-023-02861-3.

## Background

Novel coronavirus 2019 (COVID-19), caused by SARS-CoV-2, is a global pandemic that has had multiple waves [1], and several variants of concern (VOCs) with global public health significance have emerged during the pandemic [2]. Given the high cost of relying completely on non-pharmaceutical interventions (NPIs), vaccination is an important pandemic response measure [3]. Several SARS-CoV-2 vaccines have received World Health Organization (WHO) Emergency Use Listing [4], including two China-produced inactivated vaccines, BBIBP-CorV (by Sinopharm, Beijing Institute of Biological Products Co., Ltd.) and CoronaVac (by Sinovac Life Sciences Co., Ltd.). These were the first inactivated SARS-CoV-2 vaccines developed and are based on the wild‐type (WT) strain [5]; their quality, safety, and efficacy were shown in clinical trials to meet the WHO SARS-CoV-2 vaccines target product profile. Both for BBIBP-CorV and CoronaVac, two doses should be administered for primary immunization, and a booster dose may be considered 4–6 months after completion of the primary series, either heterologous or homologous doses can be used [6, 7]. Inactivated vaccines have been widely used in many countries since the earliest days of SARS-CoV-2 vaccine availability [8–10], systematic reviews and meta-analyses have reported real-world effectiveness of inactivated SARS-CoV-2 vaccines in certain periods, showing good effectiveness and acceptability generally [9, 11–14].

A growing number of studies are showing that vaccine effectiveness (VE) is influenced by factors such as time since vaccination, variants, vaccination strategies and number of doses [15–18]. However, there is currently a lack of meta-analysis of the effectiveness changes of inactivated vaccines after long-term vaccination, as well as the combined effect of related factors, no available meta-analysis perform meta-regression of multiple factors related to inactivated VE, making it difficult to explain whether and how a factor actually contributes to VE when there are other related factors, the influence of multiple factors on VE is not well understood [19–21].

Knowledge of VE and its influencing factors can help policymakers manage and adjust vaccination strategies, but additional evidence on inactivated VE is needed. We therefore conducted a systematic review with meta-analysis and multiple meta-regression to refine the evidence of effectiveness and related factors of primary series and homologous booster doses of inactivated COVID-19 VE against SARS-CoV-2 infection and severe COVID-19.

## Methods

Our systematic review with meta-analysis followed the Meta-analysis of Observational Studies in Epidemiology (MOOSE) guidelines [22]; Additional file 1: Table S1 shows the MOOSE Checklist. The study is registered with PROSPERO, registration number CRD42022353272.

### Data sources and search strategy

We searched without language restriction for studies published or preprinted by 31 August 2022 on inactivated SARS-CoV-2 vaccine efficacy or effectiveness in PubMed, Embase, Scopus, Web of Science, medRxiv, BioRxiv, and the WHO COVID-19 database VIEW-HUB website [23], which compiles searches of more than 100 databases. We searched for studies with multiple variations of the primary key search terms: [(“Effectiveness” OR “Efficacy” OR “Evaluation”) AND (COVID-19 OR SARS-CoV-2 OR Coronavirus) AND (Vaccine OR Vaccination) AND (CoronaVac or Vero Cell or BBIBP or WIBP or Inactivated). The full search strategy is shown in Additional file 1: Table S2. Additionally, the reference lists of the inactivated vaccine meta-analysis articles were hand-searched.

### Selection criteria

The selected studies met the following eligibility criteria: (a) observational study (prospective or retrospective cohort studies, case–control studies, and descriptive studies [mainly cross-sectional]); (b) assessing the effectiveness of inactivated SARS-CoV-2 vaccines (CoronaVac or BBIBP-CorV) to prevent SARS-CoV-2 infection or COVID-19-related hospitalization, severe/critical outcomes, and death; (c) reporting VE or related estimates from primary series or homologous booster vaccination at least 14 days after the last dose; and (d) with an unvaccinated reference group. We excluded randomized clinical trials, systematic reviews, and case series; we excluded studies that used only immunoglobulin M (IgM) antibody tests to diagnose COVID-19, serological studies, studies that did not report results of VE or estimates or data that can calculate estimates, and studies that only used a vaccinated reference group. Retrieved articles were exported to EndNote Reference Library, version X9.3.

### Data extraction and quality assessment

Two independent reviewers (SX and JL) performed the data extraction and quality assessment, and discussed the discrepancies. A third investigator (HW) resolved the remaining discrepancies. We extracted and analyzed VE against SARS-CoV-2 infection and severe COVID-19. SARS-CoV-2 infection had to have been confirmed by reverse transcription-polymerase chain reaction (RT-PCR) or antigen testing in accordance with the WHO recommendations [24]; studies did not clarify the test method, but confirmed infection cases were also included. Due to differences in national policies for diagnostic testing, infection generally refers to testing after the onset of symptoms, but in countries like China that have large-scale RT-PCR testing, infection includes asymptomatic, test-positive individuals.

Estimates of COVID-19-related hospitalization, severe COVID-19 cases, COVID-19-related intensive care unit (ICU) admission, and death due to COVID-19 from articles were included in the severe COVID-19 outcome of our study. From included articles, severe COVID-19 cases generally defined according to WHO, by any of the following: (1) oxygen saturation < 90% on room air; (2) in adults, signs of severe respiratory distress (accessory muscle use, inability to complete full sentences, respiratory rate > 30; breaths per minute), and in children, very severe chest wall indrawing, grunting, central cyanosis, or presence of any other general danger signs (inability to breastfeed or drink, lethargy or reduced level of consciousness, convulsions) in addition to the signs of pneumonia [25]. Severe COVID-19 cases frequently require hospitalization and, if ventilatory support is required for acute respiratory failure, admission to the ICU. Additionally, if an article included more than one of the severe outcome subtypes and overlapped, we only chose the large-scale one to avoid double counting.

If an article specified the predominant SARS-CoV-2 variant during the study period or the specific variant in the study population, and if the variant was a VOC, we associated the reported VOC with the study estimates. If an article did not mention a predominant variant, had more than one predominant variant, or the variant was not a VOC, we classified the variant as “other.” Data in one article was of the WT strain, and the study showed that antibodies in vaccine-induced serum largely retained neutralizing response against Alpha [26], so we merged the evaluation of the WT strain with the Alpha strain data. If an article did not clearly state the time between vaccination and outcome, we estimated the duration from the start or completion of vaccination in the study setting, based on the information given in the article. Some studies reported vaccine effectiveness data on multiple groups; we combined relevant data in the same study according to the needs of meta-analysis to avoid double counting.

We extracted study data into a Microsoft Excel data extraction tool. Data were basic information, including title, first author, publication year, and study design; characteristics of the study population, including number of participants, country (for representation of regional characteristics and NPI use, we grouped countries by WHO region for the meta-analysis), population type, age range (populations were divided into different age ranges for meta-analysis, ± 5 years if the original age range was not completely specified); predominant SARS-CoV-2 variant during the study period; vaccination status, including vaccine brand, number of doses (primary [full series] and booster vaccination was defined as at least 14 days since two or three doses), and time since last dose; and vaccine effectiveness outcomes, including adjusted estimates, and their 95% confidence intervals (95% CIs).

We evaluated the risk of bias using the Newcastle–Ottawa Quality Assessment Scale for cohort and case–control studies. We assessed the quality of descriptive studies using a checklist recommended by the Agency for Healthcare Research and Quality (AHRQ) [27]. Cohort studies and case–control studies were classified as having low (7 scores), moderate (5–6 scores), or high (4 scores) risk of bias with an overall quality score of 9. For descriptive studies, we assigned values to each item of the AHRQ checklist with resulting scores that ranged from 0 to 11. We categorized these scores as low, moderate, and high risk of bias with scores of 8–11, 4–7, and 0–3, respectively.

### Statistical analysis

We used DerSimonian and Laird random-effects models to calculate pooled estimates for subgroup analyses [28]: hazard ratios (HR), rate ratios (RR), or odds ratios (OR) with 95% CIs, comparing SARS-CoV-2 infection or severe COVID-19 in primary and booster vaccinated participants against different variants by time since vaccination. For data reported as frequencies or proportions, estimates were calculated directly. Vaccine effectiveness was (1-pooled HR/RR/OR) × 100%, together with 95% CIs. For vaccine effectiveness of 100% in which 95% CIs were not estimable, or if there was no event in either group in a trial, we adjusted estimates and approximated 95% CIs using study data, adding 0.5 cases to each group [29]. Negative VEs with study bias after review were excluded. We evaluated publication bias using funnel plots, Begg’s test [30], and Egger’s test [31]. The trim and fill method was used to identify and correct funnel plot asymmetry arising from publication bias [32].

We conducted multiple meta-regression with restricted maximum likelihood (REML) estimation and Knapp-Hartung adjustment to identify the factors associated with VE after adjusting for other explanatory variables [33, 34]. We used an information theoretic approach based on Akaike’s Information Criterion with the small-sample correction (AICc) for model selection, a valid model selection method outperforms other conventional methods for multiple regression or multiple meta-regression [35, 36], models with the smaller Δ_*i*_ (the difference units between the minimum AICc value model and the *i*th model) were more likely to be the potential best models, Δ_*i*_ values close to 0 have a lot of empirical support, and in the rough range, 4–7 have considerably less support [37], simply dropping models with ∆AIC will probably discard useful models [38]. Thus, we used multimodel inference to examine all possible factors combinations [36], and selected the top potential best models based on Δ_*i*_ values ranged 0–4, then decided the best model among potential ones by the coefficient of determination *(R*^*2*^) value of meta-regression, which represents the percentage of between-study heterogeneity explained by the factors of the meta-regression model [39]. We also conducted sensitivity analysis by dropping a small fraction of data to assess the robustness of meta-regression results [40], including the estimates from the moderate risk of bias studies, and the outliers identified by Cook’s distance. Factors for model selection were converted into dummy variables, factors, and their reference groups including study region (Western Pacific Region), VOC (Alpha), time since vaccination (14–90 days), vaccine brand (BBIBP-CorV), vaccine doses (primary series), age range (18–59 years old), population type (general), and study design (cohort study). Detailed factor groups can be found in Additional file 1: Tables S3 to S4.

Analyses were performed using the Meta-Analysis of Stata v17.0 and R software v4.2.2; the “metafor,” “dplyr,” “EnvStats,” and “ggplot2” packages were used for model selection, meta-regression, and visualization. A significance level of 5% was used for subsequent analysis.

## Results

**Fig. 1:**
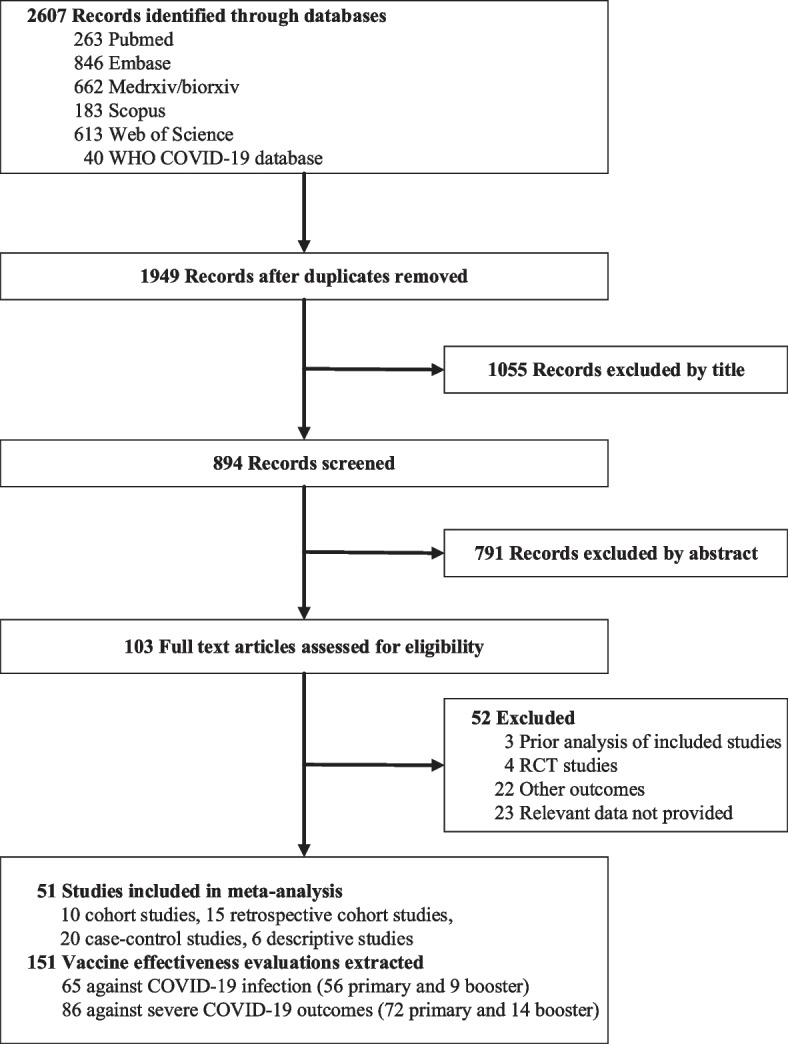
Study selection flow diagram

The initial search led to 2607 results. After deduplication and application of the eligibility criteria, 103 articles were included for full-text assessment. We discarded three redundant analyses of already-included studies; we discarded four randomized clinical trials and 45 other studies because they did not report outcomes related to vaccine effectiveness or did not provide relevant data to determine outcomes. In addition, two negative VE estimates were discarded due to study bias. Ultimately, 51 eligible studies with 151 estimates were included in the systematic review and meta-analysis. Figure 1 shows the study selection flow diagram.

### Study characteristics and quality assessment

Additional file 1: Table S5 shows the detailed characteristics of the 51 included studies [41–91], and Additional file 1: Tables S3 to S4 and S6 show the summary of the key characteristics of the included studies and VE evaluations. The total study population was 142,236,816 subjects. Among included studies, 60.78% (31/51) only used the RT-PCR test as the infection identification method, while 35.29% (18/51) used both RT-PCR and antigen test to identify infection, and 2 studies did not specify the identification method of confirmed infection cases. We extracted 151 VE evaluations, among which 65 were evaluations against SARS-CoV-2 infection and 86 were against severe COVID-19.

The quality assessment showed that the risk of bias in most of the studies (48 studies) was low. The other three studies had a moderate risk of bias. Additional file 1: Tables S7 to S9 shows the quality assessment results.

### Primary series vaccine effectiveness

Meta-analysis of 44 evaluations of primary series VE against SARS-CoV-2 infection and 57 evaluations of primary series VE against severe COVID-19 during Alpha, Gamma, Delta, and Omicron periods showed that VE against infection varied by VOC and time since vaccination and that VE against severe COVID-19 was higher than against infection and always greater than 60% against each variant (Tables 1 and 2).

**Table 1:** Primary series vaccine effectiveness against VOC

VOC	No. of estimates	Pooled estimate (95% CI)	Pooled VE (%) (95% CI)
**Against SARS-CoV-2 infection**			
Alpha	2	0.280 (0.062, 1.273)	72.0 (− 27.3, 93.8)
Gamma	7	0.558 (0.514, 0.606)	44.2 (39.4, 48.6)
Delta	26	0.472 (0.393, 0.567)	52.8 (43.3, 60.7)
Omicron	9	0.836 (0.772, 0.905)	16.4 (9.5, 22.8)
**Against severe COVID-19**			
Alpha	5	0.160 (0.059, 0.430)	84.0 (57.0, 94.1)
Gamma	7	0.267 (0.202, 0.353)	73.3 (64.7, 79.8)
Delta	31	0.306 (0.254, 0.369)	69.4 (63.1, 74.6)
Omicron	14	0.340 (0.299, 0.386)	66.0 (61.4, 70.1)

**Table 2:** Duration of primary series vaccine effectiveness against VOC

VOC	Time since vaccination	No. of estimates	Pooled estimate (95% CI)	Pooled VE (%) (95% CI)
**Against SARS-CoV-2 infection**				
Alpha	14–90	1	0.130 (0.120, 0.140)	87.0 (86.0, 88.0)
	91–180	–	–	–
	> 180	–	–	–
Gamma	14–90	4	0.538 (0.470, 0.616)	46.2 (38.4, 53.0)
	91–180	1	0.579 (0.535, 0.627)	42.1 (37.3, 46.5)
	> 180	–	–	–
Delta	14–90	5	0.437 (0.309, 0.618)	56.3 (38.2, 69.1)
	91–180	5	0.778 (0.637, 0.951)	22.2 (4.9, 36.3)
	> 180	4	0.791 (0.681, 0.919)	20.9 (8.1, 31.9)
Omicron	14–90	3	0.659 (0.572, 0.760)	34.1 (24.0, 42.8)
	91–180	–	–	–
	> 180	3	0.928 (0.883, 0.977)	7.2 (2.3, 11.7)
**Against severe COVID-19**				
Alpha	14–90	2	0.100 (0.085, 0.117)	90.0 (88.3, 91.5)
	91–180	–	–	–
	> 180	–	–	–
Gamma	14–90	3	0.225 (0.115, 0.439)	77.5 (56.1, 88.5)
	91–180	1	0.230 (0.209, 0.253)	77.0 (74.7, 79.1)
	> 180	–	–	–
Delta	14–90	7	0.213 (0.112, 0.404)	78.7 (59.6, 88.8)
	91–180	7	0.438 (0.320, 0.600)	56.2 (40.0, 68.0)
	> 180	4	0.432 (0.290, 0.643)	56.8 (35.7, 71.0)
Omicron	14–90	3	0.401 (0.299, 0.539)	59.9 (46.1, 70.1)
	91–180	–	–	–
	> 180	3	0.355 (0.281, 0.448)	64.5 (55.2, 71.9)

The pooled estimate of VE against SARS-CoV-2 infection was higher against Delta (52.8% [95% CI, 43.3 to 60.7%]) and Gamma (44.2% [95% CI, 39.4 to 48.6%]) than against Omicron (16.4% [95% CI, 9.5 to 22.8%]). VE 14–90 days after vaccination was 87.0% (95% CI, 86.0 to 88.0%) against Alpha; 46.2% (95% CI, 38.4 to 53.0%) against Gamma, remaining stable during 90–180 days (42.1% [95% CI, 37.3 to 46.5%]); 56.3% (95% CI, 38.2 to 69.1%) against Delta, decreasing to 22.2% (95% CI, 4.9 to 36.3%) and 20.9% (95% CI, 8.1 to 31.9%) beyond 90 and 180 days respectively; and 34.1% (95% CI, 24.0 to 42.8%) against Omicron, decreasing to 7.2% (95% CI, 2.3 to 11.7%) after 180 days.

The pooled estimate of VE against severe COVID-19 was 84.0% (95% CI, 57.0 to 94.1%) against Alpha, similar to VEs against Gamma (73.3% [95% CI, 64.7 to 79.8%]), Delta (69.4, [95% CI, 63.1 to 74.6%]), and Omicron (66.0% [95% CI, 61.4 to 70.1%]). VE against severe COVID-19 14–90 days after vaccination was 90.0% (95% CI, 88.3 to 91.5%) against Alpha; 77.5% (95% CI, 56.1 to 88.5%) against Gamma, remaining stable during 90–180 days after vaccination (77.0% [95% CI, 74.7 to 79.1%]); 78.7% (95% CI, 59.6 to 88.8%) against Delta, 56.2% (95% CI, 40.0 to 68.0%) and 56.8% (95% CI, 35.7 to 71.0%) beyond 90 and 180 days respectively; 59.9% (95% CI, 46.1 to 70.1%) for Omicron, remaining stable after 180 days post-vaccination (64.5% [95% CI, 55.2 to 71.9%]). More detailed results of the subgroup analysis are shown in Additional file 1: Tables S10 to S12.

### Booster dose vaccine effectiveness

Nine evaluations of booster dose VE against SARS-CoV-2 infection showed that VE was 65.2% (95% CI, 48.3 to 76.6%) against Delta, similar to the primary series VE against Delta in 14–90 days. Booster dose VE decreased to 20.3% (95% CI, 10.5 to 28.0%) against Omicron, similar to the primary series VE against Omicron in 14–90 days.

**Fig. 2:**
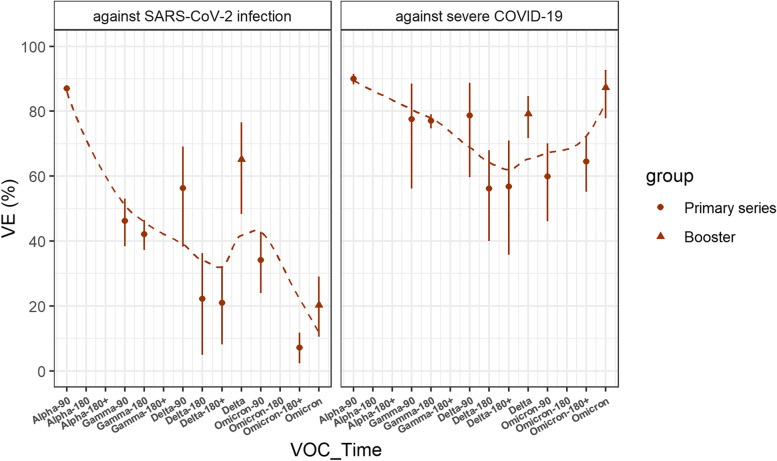
Duration of vaccine effectiveness against each variant of concern. Points with error bars are VEs and 95% CIs of primary series and booster doses against different VOC and time since vaccination. “VOC_Time” includes days since the last dose vaccination during each variant of concern, “ − 90” means 14–90 days, “ − 180” means 91–180 days, and “ − 180 + ” means ≥ 180 days. Dotted lines were the LOESS smoothing curves, indicating VE variation tendency. Effectiveness of inactivated SARS-CoV-2 vaccines for the primary series against SARS-CoV-2 infection waned over time since vaccination and VOC and was restored by booster doses during Delta and Omicron periods; effectiveness against severe COVID-19 was much greater compared to the effectiveness against infection, improved when boosted during Delta and Omicron periods

In 14 evaluations of booster dose VE against severe COVID-19, VE was 79.2% (95% CI, 71.7 to 84.7%) against Delta and 87.3% (95% CI, 77.8 to 92.7%) against Omicron, and booster dose was more effective than primary vaccination against Omicron variant (Table 3). Due to the later starting time of booster vaccination, no VE estimates more distant than 14 to 90 days after booster vaccination could be determined. Comparison of primary series and booster doses VE against different VOC and time since vaccination is shown in Fig. 2.

**Table 3:** Booster dose vaccine effectiveness against VOC

VOC	No. of estimates	Pooled estimate (95% CI)	Pooled VE (%) (95% CI)
**Against SARS-CoV-2 infection**			
Delta	4	0.348 (0.234, 0.517)	65.2 (48.3, 76.6)
Omicron	5	0.797 (0.710, 0.895)	20.3 (10.5, 28.0)
**Against severe COVID-19**			
Delta	4	0.208 (0.153, 0.283)	79.2 (71.7, 84.7)
Omicron	10	0.127 (0.073, 0.222)	87.3 (77.8, 92.7)

### Meta-regression analysis

For VE against SARS-CoV-2 infection, the lowest AICc value of potential models was 118.462; we selected eight top potential best models based on the ΔAICc among 2^8^ possible models (Table 4). After comparing the *R*^2^ values of potential best models, we found that model 6 was the best model with the highest *R*^2^ of 33.4%, and its ΔAICc was 3.451 compared to the lowest AICc (model 1). Variables in the best model were “study region,” “VOC,” and “time since vaccination,” and meta-regression results showed that each of these variables had a statistically significant association with vaccine effectiveness (Table 5). VE against Omicron variant was significantly different from VE against Alpha, and the risk of infection after vaccination during Omicron predominant period was 2.963 times compared with Alpha (the exponentiation of correlation coefficient exp(*b*) = 2.963, *P* = 0.021) after controlling for study region and time since vaccination. Risk of infection based on time since vaccination was not significantly different 90–180 days since vaccination (*P* = 0.067) compared to 14–90 days but was 1.772 times higher when the time since vaccination was greater than 180 days (exp(*b*) = 1.772, *P* = 0.022). Since vaccine doses were not the factor related to the change of VE against SARS-CoV-2 infection, and distance after booster vaccination for included studies could only be determined up to 90 days, meta-regression results also showed that booster doses restored VE to that seen 14 to 90 days after the primary series. VE against infection in the Eastern Mediterranean region was significantly different (exp(*b*) = 0.437, *P* = 0.014) from Western Pacific Region.

**Table 4:** The top best models by ΔAICc < 4 fitted to VE against SARS-CoV-2 infection

**Model**	**Factor(s)**	**AICc**	Δ**AICc**	***R***^**2**^** (%)**
1	VOC + time since vaccination	118.462	0.000	26.9
2	VOC	120.803	2.341	17.9
3	Study region + VOC	120.921	2.459	26.1
4	VOC + time since vaccination + vaccine doses	120.945	2.483	25.3
5	VOC + time since vaccination + population type	121.829	3.367	26.8
6	Study region + VOC + time since vaccination	121.913	3.451	33.4
7	VOC + population type	122.294	3.832	17.6
8	VOC + vaccine doses	122.301	3.839	17.3

**Table 5:** Meta-regression analysis of factors associated with VE against SARS-CoV-2 infection

**Factors**	***b***^a^	**95% CI**	***P***	**S.E**	**Exp(*b*)**	**95% CI**
**Study region**^**b**^						
Western Pacific Region	Ref	–	–	–	1	–
Region of Americas	− 0.110	(− 0.540, 0.320)	0.610	0.214	0.896	(0.583, 1.377)
European region	− 0.002	(− 0.494, 0.490)	0.993	0.245	0.998	(0.610, 1.632)
Region of South-East Asia	− 0.041	(− 0.673, 0.592)	0.898	0.315	0.960	(0.510, 1.808)
Eastern Mediterranean region	− 0.828	(− 1.480, − 0.175)	0.014	0.325	0.437	(0.228, 0.839)
**VOC**						
Alpha	Ref	–	–	–	1	–
Gamma	0.759	(− 0.210, 1.727)	0.122	0.483	2.136	(0.811, 5.626)
Delta	0.413	(− 0.417, 1.244)	0.323	0.414	1.512	(0.659, 3.469)
Omicron	1.086	(0.169, 2.004)	0.021	0.457	2.963	(1.184, 7.416)
Others^c^	0.543	(− 0.364, 1.451)	0.235	0.453	1.722	(0.695, 4.269)
**Time since vaccination**						
14–90	Ref	–	–	–	1	–
91–180	0.533	(− 0.039, 1.106)	0.067	0.176	1.704	(0.961, 3.021)
> 180	0.572	(0.085, 1.059)	0.022	0.285	1.772	(1.089, 2.885)
Others^d^	0.158	(− 0.194, 0.510)	0.372	0.243	1.171	(0.824, 1.666)
**Constant**	− 1.370	(− 2.258, − 0.481)	0.003	0.443	0.254	(0.105, 0.618)

*S.E *Standard error, *Exp(b)* Exponentiation of *b*, *ref* Reference group

^a^Regression coefficient

^b^Countries included in each region: Western Pacific Region includes China (Mainland and Hong Kong) and Malaysia; region of Americas includes Argentina, Brazil, Chile, Mexico, and Peru; European region includes Hungary, Kazakhstan, Serbia, and Turkey; region of South-East Asia includes Thailand and Indonesia; Eastern Mediterranean region includes Egypt, Iran, and Pakistan

^c^Mixed, no VOC or unspecified predominant variant

^d^Mixed, at least 14 days after vaccination and time interval cross groups or not extractable

For VE against severe COVID-19, the lowest AICc value was 169.355 among 2^8^ possible models; we selected three top potential best models based on the ΔAICc (Table 6). Model 1 was the best model with the highest *R*^2^ of 56.9% and the lowest AICc. Variables in the best model were “vaccine doses,” “age range,” “study design,” “study region,” “population type,” and “VOC,” and they all had a statistically significant association with VE against severe COVID-19 (Table 7). After controlling for other explanatory variables, the risk of severe COVID-19 after the booster dose was decreased (exp(*b*) = 0.541, *P* = 0.001) compared with the primary series. Compared with Alpha, risks of severe COVID-19 after vaccination during Gamma (exp(*b*) = 2.195, *P* = 0.034), Delta (exp(*b*) = 2.672, *P* = 0.001), and Omicron (exp(*b*) = 3.121, *P* = 0.001) were higher. VE against severe COVID-19 in Western Pacific Region was significantly different from the region of the Americas (exp(*b*) = 1.589, *P* = 0.034) and the European region (exp(*b*) = 2.587, *P* < 0.001).

**Table 6:** The top best models by ΔAICc < 4 fitted to VE against severe COVID-19 outcomes

**Model**	**Factors**	**AICc**	Δ**AICc**	***R***^**2**^** (%)**
1	Study region + VOC + population type + age range + vaccine doses + study design	169.355	0.000	56.9
2	Study region + population type + age range + vaccine doses + study design	172.438	3.083	46.7
3	Study region + vaccine brand + age range + vaccine doses + study design	172.552	3.197	45.3

**Table 7:** Meta-regression analysis of factors associated with VE against severe COVID-19

Factors	*b*	95% CI	*P*	S.E	Exp(*b*)	95% CI
**Study region**^**a**^						
Western Pacific region	Ref	–	–	–	1	–
Region of Americas	0.463	(0.035, 0.891)	0.034	0.214	1.589	(1.036, 2.438)
European region	2.587	(1.757, 3.418)	< 0.001	0.416	13.290	(5.795, 30.508)
Region of South-East Asia	0.077	(− 0.663, 0.816)	0.837	0.370	1.080	(0.515, 2.261)
Eastern Mediterranean region	− 0.001	(− 0.505, 0.502)	0.996	0.252	0.999	(0.604, 1.652)
**VOC**						
Alpha	Ref	–	–	–	1	–
Gamma	0.786	(0.063, 1.510)	0.034	0.362	2.195	(1.065, 4.527)
Delta	0.983	(0.413, 1.553)	0.001	0.286	2.672	(1.511, 4.726)
Omicron	1.138	(0.479, 1.798)	0.001	0.330	3.121	(1.614, 6.038)
Others	0.415	(− 0.283, 1.112)	0.240	0.350	1.514	(0.754, 3.040)
**Population type**						
General	Ref	–	–	–	1	–
HCWs	0.757	(− 0.717, 2.230)	0.309	0.738	2.132	(0.488, 9.300)
COVID-19 inpatient	1.346	(0.738, 1.955)	< 0.001	0.305	3.842	(2.092, 7.064)
Chronical patient	0.512	(− 0.147, 1.171)	0.126	0.330	1.669	(0.863, 3.225)
**Age range**						
18–59	Ref	–	–	–	1	–
< 18	− 0.546	(− 1.184, 0.093)	0.093	0.320	0.579	(0.306, 1.097)
≥ 60	0.568	(0.290, 0.847)	< 0.001	0.140	1.765	(1.336, 2.333)
Others^b^	0.503	(− 0.180, 1.186)	0.146	0.342	1.654	(0.835, 3.274)
**Vaccine doses**						
Primary	Ref	–	–	–	1	–
Booster	− 0.614	(− 0.972, − 0.257)	0.001	0.179	0.541	(0.378, 0.773)
**Study design**						
Cohort study^c^	Ref	–	–	–	1	–
Retrospective cohort study	0.883	(0.447, 1.319)	< 0.001	0.218	2.418	(1.564, 3.740)
Case–control study	0.103	(− 0.290, 0.496)	0.603	0.197	1.108	(0.748, 1.642)
Descriptive study	− 1.863	(− 2.685, − 1.040)	< 0.001	0.412	0.155	(0.068, 0.353)
**Constant**	− 2.789	(− 3.549, − 2.029)	< 0.001	0.381	0.061	(0.029, 0.131)

^a^Countries included in each region: Western Pacific Region includes China (Mainland and Hong Kong) and Malaysia; region of Americas includes Argentina, Brazil, Chile, Colombia, and Mexico; European region includes Hungary, Serbia, and Turkey; region of South-East Asia includes Thailand and Indonesia; Eastern Mediterranean region includes Egypt, Iran, Morocco, Pakistan, and United Arab Emirates

^b^Mixed, aged 18 years and older and cross-age ranges or not extractable

^c^Prospective cohort

### Publication bias and sensitivity analysis

Based on visual inspection of the funnel plot (Additional file 1: Figs. S1 to S2), and the results of Egger’s test for small-study effect, we found asymmetry for VE against SARS-CoV-2 infection (*P* < 0.001) and severe COVID-19 (*P* = 0.008). However, the results of Begg’s test for small-study effect showed no significant publication bias against SARS-CoV-2 infection (*P* = 0.865) or severe COVID-19 (*P* = 0.351). The results did not change after a trim and fill test, indicating that the impact of bias was likely not significant.

After sensitivity analysis (Additional file 1: Tables S13 to S16), meta-regression results did not change except for the model of VE against SARS-CoV-2 infection, factor “study region” related to the VE before it became insignificant after deleting the moderate risk of bias estimate, but results of other factors did not overturn or reverse, indicating the overall robustness of our meta-regression results.

## Discussion

This comprehensive systematic review and meta-regression of the effectiveness and associated factors of two globally prominent inactivated vaccines (CoronaVac and BBIBP-CorV) included 151 VE estimates in 51 studies conducted over multiple countries and included a combined total of more than 140 million subjects. Quality assessment and publication bias testing showed relatively high reliability of meta-analysis results. The two negative VE estimates against COVID-19 infection that we excluded have study bias shown in the articles, one is due to health-seeking bias in low- and middle-income populations that would increase the frequency of disease among vaccinated [75], and the other one is due to the generally more active and higher rate of social contact and frequent hospital or hemodialysis center visits for vaccinated younger patients with chronic kidney disease [89]. *R*^2^ values in meta-regression were above 30% for regressions on VE against SARS-CoV-2 infection and 50% against severe COVID-19, suggesting that the variables included in the regression model that were possible to extract from articles provided a relatively high degree of explanation of VE heterogeneity. We found that VE against COVID-19 did not vary by vaccine brand, but varied by study region and VOC. Time since vaccination associated with VE against infection specifically, while vaccine doses, age, study design, and study population type associated with VE against severe outcomes specifically.

Changes in VE against variants were observed in the meta-analysis, but for primary series VE against infection during Alpha variant predominant period, we got the opposite result (VE = 72.0%, 95% CI, − 27.3 to 93.8%) after merging two studies which both concluded that VE against Alpha had positive effectiveness. This was due to the use of a randomized effect model [92] to account for the high heterogeneity (*P* < 0.001, *I*^2^ = 99.11%) from much better VE in Petrović’s study [76] compared to Can’s [55]. In our opinion, inactivated vaccines should still have positive and high effectiveness against Alpha, since the risk of bias in two studies was low, and we found that VE was significantly decreased against Omicron compared to Alpha against SARS-CoV-2 infection in meta-regression analysis. We confirmed that VOC related to the decrease of VE, especially during the Omicron variant-predominant period against infection and severe COVID-19, and Gamma and Delta variants also related to the decrease of VE against severe COVID-19 compared to Alpha variant. These findings are consistent with recent studies showing that inactivated VE was significantly lower against some variants, especially Omicron [12, 93–96], which may be related to immune escape when comparing immunogenicity against the ancestral strain of the virus [97–99].

The risk of SARS-CoV-2 infection rose when the time since vaccination was longer than 6 months, 1.772 times that of the 14–90-day risk of infection, consistent with immunological data showing decreased antibody levels over time for different types of vaccines [95, 100–102]. However, meta-regression showed no significant difference in VE against severe COVID-19 for more than 6 months. This finding reflects longer-lasting protection provided by inactivated vaccines against severe COVID-19 compared to protection from SARS-CoV-2 infection and is similar to previous studies and meta-analysis [12, 103, 104].

VE of booster dose significantly increased compared to primary vaccination against severe COVID-19, but booster dose VE against SARS-CoV-2 infection did not change significantly compared to primary series VE when adjusting for factors such as time since vaccination. This indicates that booster doses restored effectiveness to that seen shortly after completion of the primary series (and before primary series VE waned). A caveat is that booster dose VE duration was only evaluated for a limited time, up to 90 days could be determined. Our finding is consistent with evidence from an immune response study showing that inactivated vaccine booster doses enhanced seroconversion and neutralizing capability against Delta and Omicron [105], reinforcing the necessity of booster doses 6 months after primary series vaccination.

Regional differences in VE against SARS-CoV-2 infection and severe COVID-19 are likely related to variations in prevention and control strategies, vaccination policies, and force of infection in different countries. Vaccination is an important component of the pandemic response, but vaccination alone is an incomplete response to COVID-19; public health and social measures are necessary to continue building population immunity with SARS-CoV-2 vaccines [106].

Our findings have important policy implications for SARS-CoV-2 vaccination. Inactivated vaccines have strong, sustained protection from severe COVID-19 in our meta-analysis, more than 180 days after the primary series, and VE against severe COVID-19 during Omicron predominant period was 66.0% for the primary series and 87.3% for a booster dose in the real-world studies, supporting the continued use of inactivated COVID-19 vaccines to prevent severe COVID-19. In particular, countries should keep promoting booster dose vaccination, which can restore effectiveness to that seen shortly after the completion of the primary series against SARS-CoV-2 infection, and related to the greater VE compared to the primary series against severe COVID-19. Additionally, attention should be given to the time after vaccination since primary series VE waned after 6 months against infection, and continued surveillance of booster dose VE with time is needed.

Our study has limitations. The definition of infection varies by country and region, and some studies included asymptomatic, infected individuals in the study population, which may lead to an underestimation of vaccine effectiveness. Factors such as vaccination coverage level and COVID-19 prevention and control measures may be associated with SARS-CoV-2 exposure; this information was difficult to extract from the included articles. We included study region in the meta-regression, which may alleviate the influence of control-measure variation, but it cannot eliminate related bias, and it appears to be susceptible to the change of sample due to the variation in the number of included studies across countries, as what we observed in the sensitivity analysis. Although we attempted to determine accurately the predominate VOC in the studies, the mutation of the SARS-CoV-2 virus is a gradual process, some of which included unknown mixtures of other variants and were not possible to separate. In addition, though we discarded redundant studies, it is still possible that there are similar populations among included studies, so the combined total of subjects in our included studies might be slightly lower than the number we counted.

## Conclusions

Inactivated SARS-CoV-2 vaccines were found to have moderate primary series VE against SARS-CoV-2 infection that wanes over 6 months but is restored by booster doses. Inactivated primary series VE against severe COVID-19 (hospitalization or worse) was much greater than VE against infection and sustained for more than 6 months and was highest when boosted. VE against SARS-CoV-2 infection and severe COVID-19 varied by variant, most notably waned against Omicron. These findings demonstrate optimal protection from severe COVID-19 requires booster doses, and it is important to continue monitoring the evolution of the virus and the effectiveness of the vaccines against new variants and to accelerate vaccine development to produce vaccines capable of blocking infection and preventing severe COVID-19 against a wider range of coronaviruses, either variant-specific vaccines or pan-sarbecovirus vaccines. It is also necessary to monitor the severity of breakthrough infections to ensure that vaccine protection from severe COVID-19 remains robust.

## Supplementary Information

**Additional file 1: Table S1.** MOOSE Checklist for Meta-analyses of Observational Studies. **Table S2.** Full search strategy for studies. **Table S3.** Characteristics of included studies. **Table S4.** Characteristics of VE evaluations. **Table S5.** Data extracted by included studies. **Table S6.** Population and SARS-CoV-2 infection Identification of included studies. **Table S7.** Quality assessment of cohort study. **Table S8.** Quality assessment of case-control study. **Table S9.** Quality assessment of descriptive study. **Table S10.** Subgroup analysis of primary series VE against VOC. **Table S11.** Subgroup analysis of primary series VE by time since vaccination. **Table S12.** Subgroup analysis of booster VE against VOC. **Fig. S1.** Funnel plot for VE against SARS-CoV-2 infection. **Fig. S2.** Funnel plot for VE against severe COVID-19. **Table S13.** Sensitivity analysis: meta-regression of VE against SARS-CoV-2 infection. **Table S14.** Sensitivity analysis: meta-regression of VE against severe COVID-19. **Table S15.** Sensitivity analysis: meta-regression of VE against SARS-CoV-2 infection. **Table S16.** Sensitivity analysis: meta-regression of VE against severe COVID-19.

## Data Availability

All data analyzed during this study are included in this published article and its supplementary information files.

## Abbreviations

AHRQ: Agency for healthcare research and quality
AIC: Akaike’s information criterion
CI: Confidence interval
HR: Hazard ratio
ICU: Intensive care unit
IgM: Immunoglobulin M
MOOSE: Meta-analysis of Observational Studies in Epidemiology
NPI: Non-pharmaceutical intervention
OR: Odds ratio
RR: Rate ratio
RT-PCR: Reverse transcription-polymerase chain reaction
VE: Vaccine effectiveness
VOC: Variant of concern
WHO: World health organization
WT: Wild‐type
