# Supplementary material for: Real-world effectiveness and factors associated with effectiveness of inactivated SARS-CoV-2 vaccines: a systematic review and meta-regression analysis
Source: BMC Med. 2023 Apr 27;21:160. doi: 10.1186/s12916-023-02861-3 (PMC10134725; doi:10.1186/s12916-023-02861-3)
Supplement: Supplementary file 1 — Additional file 1: Table S1. MOOSE Checklist for Meta-analyses of Observational Studies. Table S2. Full search strategy for studies. Table S3. Characteristics of included studies. Table S4. Characteristics of VE evaluations. Table S5. Data extracted by included studies. Table S6. Population and SARS-CoV-2 infection Identification of included studies. Table S7. Quality assessment of cohort study. Table S8. Quality assessment of case-control study. Table S9. Quality assessment of descriptive study. Table S10. Subgroup analysis of primary series VE against VOC. Table S11. Subgroup analysis of primary series VE by time since vaccination. Table S12. Subgroup analysis of booster VE against VOC. Fig. S1. Funnel plot for VE against SARS-CoV-2 infection. Fig. S2. Funnel plot for VE against severe COVID-19. Table S13. Sensitivity analysis: meta-regression of VE against SARS-CoV-2 infection. Table S14. Sensitivity analysis: meta-regression of VE against severe COVID-19. Table S15. Sensitivity analysis: meta-regression of VE against SARS-CoV-2 infection. Table S16. Sensitivity analysis: meta-regression of VE against severe COVID-19. [file 12916_2023_2861_MOESM1_ESM.docx]

**A****dditional File 1:** **Supplementary** **Appendix**

**Real-world Effectiveness and Factors Associated with Effectiveness of Inactivated SARS-CoV-2 Vaccines: A Systematic Review and Meta-regression Analysis**

Shiyao Xu; Jincheng Li; Hongyuan Wang; Fuzhen Wang; Zundong Yin; Zhifeng Wang

**Supplementary Content**

Table S1. MOOSE Checklist for Meta-analyses of Observational Studies 2

Table S2. Full search strategy for studies 4

Table S3. Characteristics of included studies 5

Table S4. Characteristics of VE evaluations 6

Table S5. Data extracted by included studies 7

Table S6. Population and SARS-CoV-2 infection Identification of included studies 18

Table S7. Quality assessment of cohort study 20

Table S8. Quality assessment of case-control study 21

Table S9. Quality assessment of descriptive study 22

Table S10. Subgroup analysis of primary series VE against VOC 23

Table S11. Subgroup analysis of primary series VE by time since vaccination 23

Table S12. Subgroup analysis of booster VE against VOC 24

Figure S1. Funnel plot for VE against SARS-CoV-2 infection 25

Figure S2. Funnel plot for VE against severe COVID-19 25

Table S13. Sensitivity analysis (deleting moderate risk of bias data): meta-regression of VE against SARS-CoV-2 infection 26

Table S14. Sensitivity analysis (deleting moderate risk of bias data): meta-regression of VE against severe COVID-19 27

Table S15. Sensitivity analysis (deleting outliers): meta-regression of VE against SARS-CoV-2 infection 28

Table S16. Sensitivity analysis (deleting outliers): meta-regression of VE against severe COVID-19 29

# **Table S1.** **MOOSE Checklist for Meta-analyses of Observational Studies**

| **Item No** | **Recommendation** | **Reported on Page No** |
| --- | --- | --- |
| Reporting of background should include | | |
| 1 | Problem definition | 4-5 |
| 2 | Hypothesis statement | 4-5 |
| 3 | Description of study outcome(s) | 5 |
| 4 | Type of exposure or intervention used | 5 |
| 5 | Type of study designs used | 5 |
| 6 | Study population | 4-5 |
| Reporting of search strategy should include | | |
| 7 | Qualifications of searchers (eg, librarians and investigators) | 6 |
| 8 | Search strategy, including time period included in the synthesis and key words | 5-6, Additional File 1: 4 |
| 9 | Effort to include all available studies, including contact with authors | 5-6 |
| 10 | Databases and registries searched | 5 |
| 11 | Search software used, name and version, including special features used (eg, explosion) | 5 |
| 12 | Use of hand searching (eg, reference lists of obtained articles) | 6 |
| 13 | List of citations located and those excluded, including justification | 11 |
| 14 | Method of addressing articles published in languages other than English | 5 |
| 15 | Method of handling abstracts and unpublished studies | 5 |
| 16 | Description of any contact with authors | - |
| Reporting of methods should include | | |
| 17 | Description of relevance or appropriateness of studies assembled for assessing the hypothesis to be tested | 5-6 |
| 18 | Rationale for the selection and coding of data (eg, sound clinical principles or convenience) | 5-6 |
| 19 | Documentation of how data were classified and coded (eg, multiple raters, blinding and interrater reliability) | 6 |
| 20 | Assessment of confounding (eg, comparability of cases and controls in studies where appropriate) | 9 |
| 21 | Assessment of study quality, including blinding of quality assessors, stratification or regression on possible predictors of study results | 6, 10 |
| 22 | Assessment of heterogeneity | 10 |
| 23 | Description of statistical methods (eg, complete description of fixed or random effects models, justification of whether the chosen models account for predictors of study results, dose-response models, or cumulative meta-analysis) in sufficient detail to be replicated | 9-11 |
| 24 | Provision of appropriate tables and graphics | 11, Additional File 1: 4 |
| Reporting of results should include | | |
| 25 | Graphic summarizing individual study estimates and overall estimate | 12-14, Additional File 1: 23-24 |
| 26 | Table giving descriptive information for each study included | Additional File 1: 5-19 |
| 27 | Results of sensitivity testing (eg, subgroup analysis) | 16 |
| 28 | Indication of statistical uncertainty of findings | 15-16 |
| Reporting of discussion should include | | |
| 29 | Quantitative assessment of bias (eg, publication bias) | 15-16 |
| 30 | Justification for exclusion (eg, exclusion of non-English language citations) | 16 |
| 31 | Assessment of quality of included studies | 12, 16 |
| Reporting of conclusions should include | | |
| 32 | Consideration of alternative explanations for observed results | 19-20 |
| 33 | Generalization of the conclusions (ie, appropriate for the data presented and within the domain of the literature review) | 20 |
| 34 | Guidelines for future research | 20-21 |
| 35 | Disclosure of funding source | 22 |

*From*: Stroup DF, Berlin JA, Morton SC, et al, for the Meta-analysis Of Observational Studies in Epidemiology (MOOSE) Group. Meta-analysis of Observational Studies in Epidemiology. A Proposal for Reporting. *JAMA*. 2000;283(15):2008-2012. doi: 10.1001/jama.283.15.2008.

# Table S2. Full search strategy for studies

|  | **PubMed** | **Embase** | **Web of Science** | **Scopus** | **medRxiv, bioRxiv** | **WHO view-hub** |
| --- | --- | --- | --- | --- | --- | --- |
| 1 | (Effectiveness[Title/Abstract]) OR (Efficacy[Title/Abstract]) OR (Evaluation[Title/Abstract]) | (Effectiveness) OR (Efficacy) OR (Evaluation) | (Effectiveness) OR (Efficacy) OR (Evaluation) | TITLE-ABS-KEY ((Effectiveness) OR (Efficacy)) | Effect | Vaccine studies |
| 2 | (COVID-19[Title/Abstract]) OR (SARS-CoV-2[Title/Abstract]) OR (coronavirus[Title/Abstract]) | (COVID-19) OR (SARS-CoV-2) OR (Coronavirus) | (COVID-19) OR (SARS-CoV-2) OR (Coronavirus) | TITLE-ABS-KEY ((COVID-19) OR (SARS-CoV-2) OR (Coronavirus)) | Vaccine | Effectiveness Studies |
| 3 | (Vaccine[Title/Abstract]) OR (vaccination[Title/Abstract]) | (Vaccine) OR (Vaccination) | (Vaccine) OR (Vaccination) | TITLE-ABS-KEY ((Vaccine) OR (Vaccination)) | Cov | Vaccine Product: CoronaVac |
| 4 | (CoronaVac[Title/Abstract]) OR (Vero Cell[Title/Abstract]) OR (BBIBP[Title/Abstract]) OR (WIBP[Title/Abstract]) OR (Inactivated[Title/Abstract]) | (CoronaVac) OR (‘Vero Cell’) OR (BBIBP) OR (WIBP) | (CoronaVac) OR (Vero Cell) OR (BBIBP) OR (WIBP) | TITLE-ABS-KEY ((CoronaVac) OR (Inactivated) OR (BBIBP)) | Real | Vaccine Product: BBIBP-CorV |
| 5 |  |  |  |  | Coronavac | Vaccine Product: Multiple Vaccines |
| 6 |  |  |  |  | BBIBP |  |
| 7 | 1 AND 2 AND 3 AND 4 | 1 AND 2 AND 3 AND 4 | 1 AND 2 AND 3 AND 4 | 1 AND 2 AND 3 AND 4 | 1 AND 2 AND 3 AND 4 AND (5 OR 6) | 1 AND 2 AND (3 OR 4 OR 5) |

# Table S3. Characteristics of included studies

| **Variable** | **Category** | **Number of articles** |
| --- | --- | --- |
| Study design | Cohort studies | 10 |
|  | Retrospective cohort studies | 15 |
|  | Case-control studies | 20 |
|  | Descriptive studies | 6 |
| Population type | General | 37 |
|  | Healthcare workers (HCWs) | 6 |
|  | Covid-19 inpatients | 6 |
|  | Individuals with chronic diseases | 2 |
| VOC^a^ | Alpha | 5 |
|  | Gamma | 6 |
|  | Delta | 19 |
|  | Omicron | 10 |
|  | Other^b^ | 15 |
| Study region | Eastern Mediterranean Region | 8 |
|  | European Region | 6 |
|  | Region of the Americas | 24 |
|  | South-East Asia Region | 3 |
|  | Western Pacific Region | 10 |

^a^Some studies contained estimates with more than one VOC.

^b^Mixed, no VOC or unspecified predominant variant.

# Table S4. Characteristics of VE evaluations

| **Variable** | **Category** | **Number of evaluations** |
| --- | --- | --- |
| Outcome | SARS-CoV-2 infection | 65 |
|  | Severe COVID-19 | 86 |
| Vaccine brand | BBIBP-CorV | 43 |
|  | CoronaVac | 104 |
|  | Inactivated^a^ | 4 |
| Vaccine doses | Primary with 2 doses^b^ | 128 |
|  | Homologous booster dose | 23 |
| Age range | <18 years old | 7 |
|  | 18-59 years old | 35 |
|  | ≥60 years old | 37 |
|  | Other^c^ | 72 |
| Time since vaccination^d^ | 14-90 days | 39 |
|  | 91-180 days | 14 |
|  | >180 days | 14 |
|  | Other^e^ | 84 |

^a^No distinction was made between the two vaccines brand in original studies.

^b^One article mixed 1 to 3 doses, but most of the study population was vaccinated with 2 doses, we classified the subjects as receiving 2 doses.

^c^Mixed, aged 18 years and older and cross age ranges or not extractable.

^d^Time from last dose vaccination to outcome.

^e^Mixed, at least 14 days after vaccination and time interval cross groups or not extractable.

# Table S5. Data extracted by included studies

| **Study, Year** | **Country** | **Design** | **Variants** | **Subject type** | **Age** | **Brand** | **Doses** | **Days^a^** | **Outcome** | **Risk type** | **Adjusted estimate (95% CI)** |
| --- | --- | --- | --- | --- | --- | --- | --- | --- | --- | --- | --- |
| Castillo, et al., 2021 | Colombia | cohort | Mu | general | ≥60 | CoronaVac | primary | 14-150 | hospitalization without death | HR | 0.328 (0.296, 0.363) |
| Castillo, et al., 2021 | Colombia | cohort | Mu | general | ≥60 | CoronaVac | primary | 14-150 | death | HR | 0.262 (0.200, 0.344) |
| Hitchings, et al., 2021 | Brazil | case-control | Gamma | HCWs | ≥18 | CoronaVac | primary | 14-60 | infection | OR | 0.620 (0.260, 1.460) |
| Jara, et al., 2021 | Chile | cohort | Gamma and Alpha | general | ≥16 | CoronaVac | primary | 14-60 | infection | HR | 0.341 (0.334, 0.348) |
| Jara, et al., 2021 | Chile | cohort | Gamma and Alpha | general | ≥16 | CoronaVac | primary | 14-60 | hospitalization | HR | 0.125 (0.118, 0.133) |
| Ranzani, et al., 2021 | Brazil | case-control | Gamma | general | ≥70 | CoronaVac | primary | 14-90 | infection | OR | 0.530 (0.460, 0.610) |
| Ranzani, et al., 2021 | Brazil | case-control | Gamma | general | ≥70 | CoronaVac | primary | 14-90 | hospitalization | OR | 0.450 (0.370, 0.540) |
| Suah, et al., 2021 | Malaysia | retrospective cohort | Beta and Delta | general | ≥18 | CoronaVac | primary | 14-150 | admission to ICU | OR | 0.280 (0.261, 0.301) |
| Villela, et al., 2021 | Brazil | cohort | Gamma | general | 18-59 | CoronaVac | primary | 14-150 | severe/death | RR | 0.347 (0.244, 0.494) |
| Villela, et al., 2021 | Brazil | cohort | Gamma | general | ≥60 | CoronaVac | primary | 14-150 | severe/death | RR | 0.528 (0.300, 0.928) |
| Al Kaabi, et al., 2022 | United Arab Emirates | retrospective cohort | Delta | general | ≥18 | BBIBP-CorV | primary | 14-90 | hospitalization | HR | 0.582 (0.440, 0.771) |
| Al Kaabi, et al., 2022 | United Arab Emirates | retrospective cohort | Delta |  | ≥18 | BBIBP-CorV | primary | 14-90 | hospitalization | HR | 0.079 (0.009, 0.724) |
| Arregocés-Castillo, et al., 2022 | Colombia | retrospective cohort | Mu | general | ≥60 | CoronaVac | primary | 14-210 | hospitalization without death | HR | 0.527 (0.477, 0.581) |
| Arregocés-Castillo, et al., 2022 | Colombia | retrospective cohort | Mu | general | ≥60 | CoronaVac | primary | 14-210 | death | HR | 0.312 (0.249, 0.391) |
| Arriola, et al., 2022 | Peru | cohort | B.1.1.1 | HCWs | 18-65 | BBIBP-CorV | primary | 14-60 | infection | OR | 0.050 (0.010, 0.300) |
| Ashmawy, et al., 2022 | Egypt | cohort | Delta | HCWs | - | BBIBP-CorV | primary | 14-210 | infection | HR | 0.330 (0.200, 0.570) |
| Ashmawy, et al., 2022 | Egypt | cohort | Delta | HCWs | - | BBIBP-CorV | primary | 14-210 | hospitalization (severe/critical) | HR | 0.350 (0.120, 1.080) |
| Aslam, et al., 2022 | Pakistan | cohort | Delta | Covid-19 inpatients | ≥18 | Inactivated | primary | 14-120 | death | RR | 0.607 (0.482, 0.763) |
| Bayhan, et al., 2022 | Turkey | cross-sectional | Delta | HCWs | 18-59 | CoronaVac | primary | 14-180 | infection | OR | 0.059 (0.036, 0.099) |
| Belayachi, et al., 2022 | Morocco | case-control | Delta | general | ≥18 | BBIBP-CorV | primary | 31-90 | hospitalization (severe/critical) | OR | 0.130 (0.100, 0.170) |
| Belayachi, et al., 2022 | Morocco | case-control | Delta | general | ≥18 | BBIBP-CorV | primary | 90-180 | hospitalization (severe/critical) | OR | 0.315 (0.204, 0.488) |
| Bello-Chavolla, et al., 2022 | Mexico | retrospective cohort | B.1.1.519 and Delta | general | ≥18 | CoronaVac | primary | 14-240 | infection | HR | 0.281 (0.275, 0.287) |
| Bello-Chavolla, et al., 2022 | Mexico | retrospective cohort | B.1.1.519 and Delta | general | ≥18 | CoronaVac | primary | 14-240 | hospitalization | HR | 0.262 (0.250, 0.275) |
| Can, et al., 2022 | Turkey | retrospective cohort | Alpha | HCWs | 18-59 | CoronaVac | primary | 14-120 | infection | HR | 0.610 (0.463, 0.804) |
| Cerqueira-Silva, et al., 2022a | Brazil | case-control | Gamma | general | ≥18 | CoronaVac | primary | 14-90 | infection | OR | 0.595 (0.557, 0.636) |
| Cerqueira-Silva, et al., 2022a | Brazil | case-control | Gamma | general | ≥18 | CoronaVac | primary | 90-270 | infection | OR | 0.620 (0.575, 0.669) |
| Cerqueira-Silva, et al., 2022a | Brazil | case-control | Gamma | general | ≥18 | CoronaVac | primary | 14-90 | hospitalization or death | OR | 0.134 (0.097, 0.202) |
| Cerqueira-Silva, et al., 2022a | Brazil | case-control | Gamma | general | ≥18 | CoronaVac | primary | 90-270 | hospitalization or death | OR | 0.256 (0.178, 0.367) |
| Cerqueira-Silva, et al., 2022b | Brazil | case-control | Omicron | general | ≥18 | CoronaVac | primary | 14-69 | infection | OR | 0.766 (0.717, 0.818) |
| Cerqueira-Silva, et al., 2022b | Brazil | case-control | Omicron | general | ≥18 | CoronaVac | primary | 14-69 | hospitalization or death | OR | 0.659 (0.337, 1.289) |
| Cerqueira-Silva, et al., 2022b | Brazil | case-control | Omicron | general | ≥18 | CoronaVac | primary | ≥70 | hospitalization or death | OR | 0.638 (0.533, 0.765) |
| Cerqueira-Silva, et al., 2022b | Brazil | case-control | Omicron | general | ≥18 | CoronaVac | primary | ≥70 | infection | OR | 0.901 (0.854, 0.952) |
| Cerqueira-Silva, et al., 2022c | Brazil | case-control | Omicron | general | ≥18 | CoronaVac | primary | 14-180 | infection | OR | 1.007 (0.998, 1.016) |
| Cerqueira-Silva, et al., 2022c | Brazil | case-control | Omicron | general | ≥18 | CoronaVac | primary | > 180 | infection | OR | 0.968 (0.958, 0.979) |
| Cerqueira-Silva, et al., 2022c | Brazil | case-control | Omicron | general | ≥18 | CoronaVac | primary | 14-180 | hospitalization or death | OR | 0.355 (0.337, 0.374) |
| Cerqueira-Silva, et al., 2022c | Brazil | case-control | Omicron | general | ≥18 | CoronaVac | primary | > 180 | hospitalization or death | OR | 0.382 (0.368, 0.397) |
| Cerqueira-Silva, et al., 2022d | Brazil | case-control | Delta | general | ≥18 | CoronaVac | primary | >180 | infection | OR | 0.653 (0.637, 0.669) |
| Cerqueira-Silva, et al., 2022d | Brazil | case-control | Delta | general | ≥18 | CoronaVac | primary | >180 | hospitalization or death | OR | 0.274 (0.258, 0.290) |
| Cerqueira-Silva, et al., 2022d | Brazil | case-control | Delta | general | ≥18 | CoronaVac | primary | 14-90 | infection | OR | 0.485 (0.446, 0.527) |
| Cerqueira-Silva, et al., 2022d | Brazil | case-control | Delta | general | ≥18 | CoronaVac | primary | 90-180 | infection | OR | 0.579 (0.535, 0.627) |
| Cerqueira-Silva, et al., 2022d | Brazil | case-control | Delta | general | ≥18 | CoronaVac | primary | 14-90 | hospitalization or death | OR | 0.183 (0.170, 0.196) |
| Cerqueira-Silva, et al., 2022d | Brazil | case-control | Delta | general | ≥18 | CoronaVac | primary | 90-180 | hospitalization or death | OR | 0.230 (0.209, 0.253) |
| González, et al., 2022 | Argentina | retrospective cohort | Omicron | general | 3-11 | BBIBP-CorV | primary | 14-120 | hospitalization | OR | 0.236 (0.155, 0.371) |
| Institute of Public Health of Chile, 2022 | Chile | case-control | Lambda, Gamma, Delta | Covid-19 inpatients | - | CoronaVac | primary | 14-300 | infection | OR | 0.405 (0.321, 0.510) |
| Hu, et al., 2022 | China | cross-sectional | Delta | Covid-19 inpatients | 18-59 | Inactivated | primary | ≥14 | severe | OR | 0.120 (0.020, 0.610) |
| Hu, et al., 2022 | China | cross-sectional | Delta | Covid-19 inpatients | ≥60 | Inactivated | primary | ≥14 | severe | OR | 0.163 (0.009, 2.838) |
| Jara, et al., 2022a | Chile | cohort | Delta | general | 6-16 | CoronaVac | primary | 14-180 | infection | HR | 0.252 (0.245, 0.259) |
| Jara, et al., 2022a | Chile | cohort | Delta | general | 6-16 | CoronaVac | primary | 14-180 | hospitalization | HR | 0.087 (0.064, 0.119) |
| Jara, et al., 2022b | Chile | cohort | Omicron | general | 3-5 | CoronaVac | primary | 14-60 | infection | HR | 0.621 (0.604, 0.639) |
| Jara, et al., 2022b | Chile | cohort | Omicron | general | 3-5 | CoronaVac | primary | 14-60 | hospitalization | HR | 0.348 (0.244, 0.496) |
| Jara, et al., 2022c | Chile | cohort | Delta | general | ≥16 | CoronaVac | booster | 14-90 | infection | HR | 0.219 (0.201, 0.239) |
| Jara, et al., 2022c | Chile | cohort | Delta | general | ≥16 | CoronaVac | booster | 14-90 | hospitalization | HR | 0.153 (0.129, 0.182) |
| Ma, et al., 2022 | China | retrospective cohort | Delta | general | ≥18 | CoronaVac | primary | 14-150 | infection | RR | 0.270 (0.094, 0.777) |
| Ma, et al., 2022 | China | retrospective cohort | Delta | general | ≥18 | CoronaVac | primary | 14-150 | severe | RR | 0.230 (0.011, 4.850) |
| Ma, et al., 2022 | China | retrospective cohort | Delta | general | ≥18 | BBIBP-CorV | primary | 14-150 | infection | RR | 0.245 (0.064, 0.937) |
| Ma, et al., 2022 | China | retrospective cohort | Delta | general | ≥18 | BBIBP-CorV | primary | 14-150 | severe | RR | 0.283 (0.013, 5.991) |
| Marra, et al., 2022 | Brazil | retrospective cohort | Gamma | HCWs | 18-84 | CoronaVac | primary | 14-180 | infection | RR | 0.487 (0.363, 0.654) |
| McMenamin, et al., 2022 | China | ecological | Omicron | general | 20-59 | CoronaVac | primary | ≥14 | severe/death | RR | 0.083 (0.060, 0.113) |
| McMenamin, et al., 2022 | China | ecological | Omicron | general | ≥60 | CoronaVac | primary | ≥14 | severe/death | RR | 0.283 (0.189, 0.423) |
| McMenamin, et al., 2022 | China | ecological | Omicron | general | 20-59 | CoronaVac | booster | ≥14 | severe/death | RR | 0.012 (0.005, 0.025) |
| McMenamin, et al., 2022 | China | ecological | Omicron | general | ≥60 | CoronaVac | booster | ≥14 | severe/death | RR | 0.033 (0.023, 0.048) |
| Mirahmadizadeh, et al., 2022 | Iran | retrospective cohort | - | general | ≥65 | BBIBP-CorV | primary | 14-180 | infection | RR | 0.709 (0.672, 0.747) |
| Mirahmadizadeh, et al., 2022 | Iran | retrospective cohort | - | general | ≥65 | BBIBP-CorV | primary | 14-180 | hospitalization | RR | 0.543 (0.508, 0.579) |
| Mirahmadizadeh, et al., 2022 | Iran | retrospective cohort | - | general | 18-59 | BBIBP-CorV | primary | 14-180 | infection | RR | 0.144 (0.125, 0.165) |
| Mirahmadizadeh, et al., 2022 | Iran | retrospective cohort | - | general | 18-59 | BBIBP-CorV | primary | 14-180 | hospitalization | RR | 0.118 (0.087, 0.160) |
| Mousa, et al., 2022 | United Arab Emirates | case-control | Delta | general | - | BBIBP-CorV | primary | ≥14 days | hospitalization | OR | 0.050 (0.030, 0.060) |
| Nabirova, et al., 2022 | Kazakhstan | retrospective cohort | - | general | ≥18 | CoronaVac | primary | ≥14 | infection | HR | 0.305 (0.280, 0.333) |
| Nadeem, et al., 2022 | Pakistan | case-control | Delta | general | ≥60 | BBIBP-CorV | primary | 14-180 | infection | OR | 0.057 (0.041, 0.078) |
| Nadeem, et al., 2022 | Pakistan | case-control | Delta | general | ≥60 | BBIBP-CorV | primary | 14-180 | hospitalization | OR | 0.395 (0.171, 0.921) |
| O, Zolt A, et al., 2022 | Hungary | cross-sectional | Delta | general | 18-64 | BBIBP-CorV | primary | 14-120 | infection | RR | 0.891 (0.850, 0.933) |
| O, Zolt A, et al., 2022 | Hungary | cross-sectional | Delta | general | 18-64 | BBIBP-CorV | primary | 121-180 | infection | RR | 0.951 (0.930, 0.972) |
| O, Zolt A, et al., 2022 | Hungary | cross-sectional | Delta | general | 18-64 | BBIBP-CorV | booster | 14-120 | infection | RR | 0.394 (0.333, 0.466) |
| O, Zolt A, et al., 2022 | Hungary | cross-sectional | Delta | general | 65-100 | BBIBP-CorV | primary | 14-120 | infection | RR | 0.702 (0.581, 0.848) |
| O, Zolt A, et al., 2022 | Hungary | cross-sectional | Delta | general | 65-100 | BBIBP-CorV | primary | 121-180 | infection | RR | 0.746 (0.694, 0.801) |
| O, Zolt A, et al., 2022 | Hungary | cross-sectional | Delta | general | 65-100 | BBIBP-CorV | booster | 14-120 | infection | RR | 0.398 (0.320, 0.496) |
| O, Zolt A, et al., 2022 | Hungary | cross-sectional | Delta | general | 18-64 | BBIBP-CorV | primary | 14-120 | hospitalization | RR | 0.462 (0.381, 0.561) |
| O, Zolt A, et al., 2022 | Hungary | cross-sectional | Delta | general | 18-64 | BBIBP-CorV | primary | 121-180 | hospitalization | RR | 0.502 (0.461, 0.543) |
| O, Zolt A, et al., 2022 | Hungary | cross-sectional | Delta | general | 18-64 | BBIBP-CorV | booster | 14-120 | hospitalization | RR | 0.225 (0.121, 0.418) |
| O, Zolt A, et al., 2022 | Hungary | cross-sectional | Delta | general | 65-100 | BBIBP-CorV | primary | 14-120 | hospitalization | RR | 0.543 (0.421, 0.700) |
| O, Zolt A, et al., 2022 | Hungary | cross-sectional | Delta | general | 65-100 | BBIBP-CorV | primary | 121-180 | hospitalization | RR | 0.515 (0.463, 0.571) |
| O, Zolt A, et al., 2022 | Hungary | cross-sectional | Delta | general | 65-100 | BBIBP-CorV | booster | 14-120 | hospitalization | RR | 0.254 (0.173, 0.373) |
| O, Zolt A, et al., 2022 | Hungary | cross-sectional | Delta | general | 18-64 | BBIBP-CorV | primary | >180 | infection | RR | 1.118 (0.993, 1.258) |
| O, Zolt A, et al., 2022 | Hungary | cross-sectional | Delta | general | 65-100 | BBIBP-CorV | primary | >180 | infection | RR | 0.852 (0.823, 0.883) |
| O, Zolt A, et al., 2022 | Hungary | cross-sectional | Delta | general | 18-64 | BBIBP-CorV | primary | >180 | hospitalization | RR | 0.536 (0.495, 0.582) |
| O, Zolt A, et al., 2022 | Hungary | cross-sectional | Delta | general | 65-100 | BBIBP-CorV | primary | >180 | hospitalization | RR | 0.604 (0.573, 0.636) |
| Paixao, et al., 2022 | Brazil | case-control | - | general | 18-49 | CoronaVac | primary | ≥14 | infection | OR | 0.590 (0.470, 0.720) |
| Paixao, et al., 2022 | Brazil | case-control | - | general | 18-49 | CoronaVac | primary | ≥14 | severe | OR | 0.140 (0.050, 0.400) |
| Paternina-Caicedo, et al., 2022 | Colombia | retrospective cohort | Mu | general | ≥40 | CoronaVac | primary | 14-150 | hospitalization | HR | 0.967 (0.813, 1.151) |
| Petrović, et al., 2022 | Serbia | cross-sectional | Alpha | general | ≥60 | BBIBP-CorV | primary | 14-90 | infection | RR | 0.130 (0.120, 0.140) |
| Petrović, et al., 2022 | Serbia | cross-sectional | Alpha | general | ≥60 | BBIBP-CorV | primary | 14-90 | severe | RR | 0.100 (0.080, 0.110) |
| Ranzani, et al., 2022 | Brazil | case-control | Omicron | general | 18-59 | CoronaVac | primary | ≥180 | infection | OR | 0.992 (0.980, 1.004) |
| Ranzani, et al., 2022 | Brazil | case-control | Omicron | general | 18-59 | CoronaVac | primary | ≥180 | hospitalization or death | OR | 0.290 (0.247, 0.341) |
| Ranzani, et al., 2022 | Brazil | case-control | Omicron | general | 18-59 | CoronaVac | booster | 8-59 | infection | OR | 0.919 (0.886, 0.954) |
| Ranzani, et al., 2022 | Brazil | case-control | Omicron | general | 18-59 | CoronaVac | booster | 8-59 | hospitalization or death | OR | 0.140 (0.069, 0.283) |
| Ranzani, et al., 2022 | Brazil | case-control | Omicron | general | 18-59 | CoronaVac | booster | >=60 | hospitalization or death | OR | 0.180 (0.083, 0.391) |
| Ranzani, et al., 2022 | Brazil | case-control | Omicron | general | ≥60 | CoronaVac | booster | 8-59 | infection | OR | 0.748 (0.540, 1.037) |
| Ranzani, et al., 2022 | Brazil | case-control | Omicron | general | ≥60 | CoronaVac | booster | 8-59 | hospitalization or death | OR | 0.316 (0.119, 0.840) |
| Ranzani, et al., 2022 | Brazil | case-control | Omicron | general | ≥60 | CoronaVac | booster | >=60 | infection | OR | 0.901 (0.840, 0.967) |
| Ranzani, et al., 2022 | Brazil | case-control | Omicron | general | ≥60 | CoronaVac | booster | >=60 | hospitalization or death | OR | 0.342 (0.171, 0.686) |
| Ranzani, et al., 2022 | Brazil | case-control | Delta | general | ≥18 | CoronaVac | primary | 14-59 | infection | OR | 0.487 (0.473, 0.501) |
| Ranzani, et al., 2022 | Brazil | case-control | Delta | general | ≥18 | CoronaVac | primary | 60-179 | infection | OR | 0.624 (0.609, 0.649) |
| Ranzani, et al., 2022 | Brazil | case-control | Delta | general | ≥18 | CoronaVac | primary | ≥180 | infection | OR | 0.660 (0.643, 0.677) |
| Ranzani, et al., 2022 | Brazil | case-control | Delta | general | ≥18 | CoronaVac | primary | 14-59 | hospitalization or death | OR | 0.135 (0.111, 0.166) |
| Ranzani, et al., 2022 | Brazil | case-control | Delta | general | ≥18 | CoronaVac | primary | 60-179 | hospitalization or death | OR | 0.290 (0.268, 0.315) |
| Ranzani, et al., 2022 | Brazil | case-control | Delta | general | ≥18 | CoronaVac | primary | ≥180 | hospitalization or death | OR | 0.391 (0.358, 0.427) |
| Ranzani, et al., 2022 | Brazil | case-control | Omicron | general | ≥60 | CoronaVac | primary | ≥180 | infection | OR | 0.750 (0.693, 0.813) |
| Ranzani, et al., 2022 | Brazil | case-control | Omicron | general | ≥60 | CoronaVac | primary | ≥180 | hospitalization or death | OR | 0.466 (0.291, 0.749) |
| Ranzani, et al., 2022 | Brazil | case-control | Delta | general | ≥18 | CoronaVac | booster | 8-89 | infection | OR | 0.433 (0.378, 0.496) |
| Ranzani, et al., 2022 | Brazil | case-control | Delta | general | ≥18 | CoronaVac | booster | 8-89 | hospitalization or death | OR | 0.241 (0.185, 0.314) |
| Rearte, et al., 2022 | Argentina | case-control | - | general | ≥60 | BBIBP-CorV | primary | 14-180 | infection | OR | 0.560 (0.550, 0.580) |
| Rearte, et al., 2022 | Argentina | case-control | - | general | ≥60 | BBIBP-CorV | primary | 14-180 | death | HR | 0.270 (0.250, 0.290) |
| Sritipsukho, et al., 2022 | Thailand | case-control | Delta | Covid-19 inpatients | ≥18 | CoronaVac | primary | 14-180 | infection | OR | 0.400 (0.310, 0.510) |
| Suah, et al., 2022 | Malaysia | retrospective cohort | Delta | general | ≥15 | CoronaVac | primary | 14-90 | infection | RR | 0.255 (0.220, 0.294) |
| Suah, et al., 2022 | Malaysia | retrospective cohort | Delta | general | ≥15 | CoronaVac | primary | 90-180 | infection | RR | 0.696 (0.597, 0.812) |
| Suah, et al., 2022 | Malaysia | retrospective cohort | Delta | general | ≥15 | CoronaVac | primary | 14-90 | admission to ICU | RR | 0.440 (0.398, 0.488) |
| Suah, et al., 2022 | Malaysia | retrospective cohort | Delta | general | ≥15 | CoronaVac | primary | 90-180 | admission to ICU | RR | 0.713 (0.578, 0.877) |
| Suphanchaimat, et al., 2022 | Thailand | case-control | Delta | general | 18-59 | CoronaVac | primary | 90-270 | infection | OR | 0.502 (0.484, 0.522) |
| Suphanchaimat, et al., 2022 | Thailand | case-control | Delta | general | 18-59 | CoronaVac | primary | 90-270 | severe/death | OR | 0.197 (0.138, 0.280) |
| Suphanchaimat, et al., 2022 | Thailand | case-control | Delta | general | 18-59 | CoronaVac | primary | 14-90 | infection | OR | 0.504 (0.416, 0.611) |
| Suphanchaimat, et al., 2022 | Thailand | case-control | Delta | general | 18-59 | CoronaVac | primary | 14-90 | severe/death | OR | 0.116 (0.062, 0.216) |
| Suryatma, et al., 2022 | Indonesia | case-control | before Delta | general | ≥18 | CoronaVac | primary | 14-150 | infection | OR | 0.330 (0.260, 0.420) |
| Suryatma, et al., 2022 | Indonesia | case-control | before Delta | general | ≥18 | CoronaVac | primary | 14-150 | hospitalization | OR | 0.290 (0.220, 0.370) |
| Toker, et al., 2022 | Turkey | cross-sectional | Alpha | Covid-19 inpatients | ≥16 | CoronaVac | primary | ≥14 | death | OR | 0.456 (0.283, 0.732) |
| Torres, et al., 2022 | Chile | cohort | - | Covid-19 inpatients | ≥18 | CoronaVac | primary | 14-180 | infection | HR | 0.850 (0.786, 0.917) |
| Torres, et al., 2022 | Chile | cohort | - | Covid-19 inpatients | ≥18 | CoronaVac | primary | 14-180 | hospitalization | HR | 0.599 (0.517, 0.680) |
| Wu, et al., 2022 | China | retrospective cohort | Delta | general | ≥18 | BBIBP-CorV | primary | 14-90 | infection | RR | 0.606 (0.305, 1.204) |
| Wu, et al., 2022 | China | retrospective cohort | Delta | general | ≥18 | BBIBP-CorV | primary | 14-90 | severe | RR | 0.129 (0.008, 2.170) |
| Wu, et al., 2022 | China | retrospective cohort | Delta | general | ≥18 | BBIBP-CorV | primary | 90-180 | infection | RR | 0.180 (0.026, 1.257) |
| Wu, et al., 2022 | China | retrospective cohort | Delta | general | ≥18 | BBIBP-CorV | primary | 90-180 | severe | RR | 0.250 (0.015, 4.228) |
| Wu, et al., 2022 | China | retrospective cohort | Delta | general | ≥18 | CoronaVac | primary | 14-90 | infection | RR | 0.545 (0.280, 1.060) |
| Wu, et al., 2022 | China | retrospective cohort | Delta | general | ≥18 | CoronaVac | primary | 14-90 | severe | RR | 0.105 (0.006, 1.762) |
| Wu, et al., 2022 | China | retrospective cohort | Delta | general | ≥18 | CoronaVac | primary | 90-180 | infection | RR | 0.702 (0.349, 1.411) |
| Wu, et al., 2022 | China | retrospective cohort | Delta | general | ≥18 | CoronaVac | primary | 90-180 | severe | RR | 0.132 (0.008, 2.217) |
| Zhang, et al., 2022 | Morocco | case-control | Alpha | general | ≥60 | BBIBP-CorV | primary | ≥14 | severe | OR | 0.467 (0.361, 0.604) |
| Zhang, et al., 2022 | Morocco | case-control | Alpha | general | 18-59 | BBIBP-CorV | primary | ≥14 | severe | OR | 0.033 (0.012, 0.090) |
| Florentino, et al., 2022 | Brazil | case-control | Omicron | general | 6-11 | CoronaVac | primary | 14-90 | infection | OR | 0.600 (0.550, 0.660) |
| Florentino, et al., 2022 | Brazil | case-control | Omicron | general | 6-11 | CoronaVac | primary | 14-90 | hospitalization | OR | 0.410 (0.160, 0.890) |
| Yan, et al., 2022 | China | case-control | Omicron | general | ≥65 | CoronaVac | primary | 14-360 | severe | OR | 0.433 (0.356, 0.526) |
| Yan, et al., 2022 | China | case-control | Omicron | general | ≥65 | CoronaVac | booster | 14-360 | severe | OR | 0.164 (0.099, 0.270) |
| Yan, et al., 2022 | China | case-control | Omicron | general | 18-59 | CoronaVac | primary | 14-360 | severe | OR | 0.309 (0.207, 0.460) |
| Yan, et al., 2022 | China | case-control | Omicron | general | 18-59 | CoronaVac | booster | 14-360 | severe | OR | 0.162 (0.075, 0.353) |
| Tak Cheng, et al., 2022 | China | retrospective cohort | - | chronic patients | ≥65 | CoronaVac | primary | 14-360 | infection | RR | 0.970 (0.840, 1.110) |
| Tak Cheng, et al., 2022 | China | retrospective cohort | - | chronic patients | ≥65 | CoronaVac | primary | 14-360 | hospitalization | RR | 0.820 (0.460, 1.480) |
| Tak Cheng, et al., 2022 | China | retrospective cohort | - | chronic patients | <65 | CoronaVac | primary | 14-360 | hospitalization | RR | 1.350 (0.501, 3.601) |
| Tak Cheng, et al., 2022 | China | retrospective cohort | - | chronic patients | ≥18 | CoronaVac | booster | ≥14 | infection | RR | 0.390 (0.280, 0.520) |
| Tak Cheng, et al., 2022 | China | retrospective cohort | - | chronic patients | ≥18 | CoronaVac | booster | ≥14 | hospitalization | RR | 0.350 (0.130, 0.950) |
| Wan, et al., 2022 | China | case-control | Omicron | chronic patients | ≥12 | CoronaVac | primary | - | infection | OR | 1.003 (0.979, 1.027) |
| Wan, et al., 2022 | China | case-control | Omicron | chronic patients | ≥12 | CoronaVac | booster | - | infection | OR | 0.802 (0.777, 0.828) |
| Wan, et al., 2022 | China | case-control | Omicron | chronic patients | ≥12 | CoronaVac | primary | - | hospitalization | OR | 0.358 (0.336, 0.382) |
| Wan, et al., 2022 | China | case-control | Omicron | chronic patients | ≥12 | CoronaVac | booster | - | hospitalization | OR | 0.146 (0.127, 0.168) |
| Kang, et al., 2022c | China | retrospective cohort | Delta | general | ≥18 | Inactivated | primary | 14-150 | infection | RR | 0.482 (0.168, 0.797) |

^a^Time since last dose vaccination (days), estimated based on the information given in the article if not clearly stated.

# Table S6. Population and SARS-CoV-2 infection Identification of included studies

| **Study, Year** | **Population** | **Infection identification methods** | |
| --- | --- | --- | --- |
|  |  | RT-PCR | Antigen test |
| Castillo, et al.(2021) | 3346826 | √ | √ |
| Hitchings, et al.(2021) | 2147 | √ | √ |
| Jara, et al.(2021) | 10187720 | √ | √ |
| Ranzani, et al.(2021) | 43774 | √ | √ |
| Suah, et al.(2021) | 1239445 | √ | √ |
| Villela, et al.(2021) | 65210769 | √ | × |
| Al Kaabi, et al.(2022) | 2199772 | √ | × |
| Arregocés-Castillo, et al.(2022) | 2828294 | Not clarified | |
| Arriola, et al.(2022) | 290 | √ | × |
| Ashmawy, et al.(2022) | 1228 | √ | × |
| Aslam, et al.(2022) | 1514 | Not clarified | |
| Bayhan, et al.(2022) | 628 | √ | × |
| Belayachi, et al.(2022) | 25768 | √ | × |
| Bello-Chavolla, et al.(2022) | 5585825 | √ | √ |
| Can, et al.(2022) | 3174 | √ | × |
| Cerqueira-Silva, et al.(2022)a | 90992 | √ | √ |
| Cerqueira-Silva, et al.(2022)b | 899050 | √ | × |
| Cerqueira-Silva, et al.(2022)c | 2471576 | √ | √ |
| Cerqueira-Silva, et al.(2022)d | 7314318 | √ | √ |
| González, et al.(2022) | 1536435 | √ | × |
| Institute of Public Health of Chile.(2022) | 3160 | √ | × |
| Hu, et al.(2022) | 476 | √ | × |
| Jara, et al.(2022)a | 1976344 | √ | √ |
| Jara, et al.(2022)b | 490694 | √ | √ |
| Jara, et al.(2022)c | 8088863 | √ | √ |
| Ma, et al.(2022) | 686 | √ | × |
| Marra, et al.(2022) | 13813 | √ | × |
| McMenamin, et al.(2022) | 14441 | √ | × |
| Mirahmadizadeh, et al.(2022) | 1882148 | √ | × |
| Mousa, et al.(2022) | 3782 | √ | × |
| Nabirova, et al.(2022) | 1312928 | √ | × |
| Nadeem, et al.(2022) | 3426 | √ | × |
| O, Zolt A, et al.(2022) | 8087988 | √ | √ |
| Paixao, et al.(2022) | 19838 | √ | × |
| Paternina-Caicedo, et al.(2022) | 719735 | √ | √ |
| Petrović, et al.(2022) | 416448 | √ | √ |
| Ranzani, et al.(2022) | 2118094 | √ | √ |
| Rearte, et al.(2022) | 1282928 | √ | × |
| Sritipsukho, et al.(2022) | 3353 | √ | × |
| Suah, et al.(2022) | 10008348 | √ | √ |
| Suphanchaimat, et al.(2022) | 1460458 | √ | × |
| Suryatma, et al.(2022) | 5518 | √ | × |
| Toker, et al.(2022) | 559 | √ | × |
| Torres, et al.(2022) | 12301 | √ | × |
| Wu, et al.(2022) | 1462 | √ | × |
| Zhang, et al.(2022) | 348190 | √ | × |
| Florentino, et al.(2022) | 197958 | √ | √ |
| Yan, et al.(2022) | 98461 | √ | × |
| Tak Cheng, et al.(2022) | 103143 | √ | × |
| Wan, et al.(2022) | 560923 | √ | × |
| Kang, et al.(2022) | 10805 | √ | × |

# Table S7. Quality assessment of cohort study

| **Study, Year (Reference)** | **Selection^a^** | | | | **Comparability^b^** | **Outcome^c^** | | | **Total score** | **risk of bias** |
| --- | --- | --- | --- | --- | --- | --- | --- | --- | --- | --- |
|  | **A** | **B** | **C** | **D** | **A** | **A** | **B** | **C** |  |  |
| Castillo, et al., 2021 | 1 | 1 | 1 | 1 | 2 | 1 | 1 | 1 | 9 | low |
| Jara, et al., 2021 | 1 | 1 | 1 | 1 | 2 | 1 | 1 | 1 | 9 | low |
| Suah, et al., 2021 | 1 | 1 | 1 | 1 | 2 | 1 | 1 | 1 | 9 | low |
| Villela, et al., 2021 | 1 | 1 | 1 | 0 | 2 | 1 | 1 | 1 | 8 | low |
| Al Kaabi, et al., 2022 | 1 | 1 | 1 | 1 | 2 | 1 | 1 | 1 | 9 | low |
| Arregocés-Castillo, et al., 2022 | 1 | 1 | 1 | 1 | 2 | 1 | 1 | 1 | 9 | low |
| Arriola, et al., 2022 | 0 | 1 | 1 | 1 | 2 | 1 | 1 | 1 | 8 | low |
| Ashmawy, et al., 2022 | 0 | 1 | 1 | 1 | 1 | 1 | 1 | 1 | 7 | low |
| Aslam, et al., 2022 | 0 | 1 | 1 | 1 | 2 | 1 | 1 | 1 | 8 | low |
| Bello-Chavolla, et al., 2022 | 1 | 1 | 1 | 1 | 2 | 1 | 1 | 1 | 9 | low |
| Can, et al., 2022 | 0 | 1 | 1 | 1 | 2 | 1 | 0 | 1 | 7 | low |
| González, et al., 2022 | 1 | 1 | 1 | 1 | 2 | 1 | 1 | 1 | 9 | low |
| Jara, et al., 2022a | 1 | 1 | 1 | 1 | 2 | 1 | 1 | 1 | 9 | low |
| Jara, et al., 2022b | 1 | 1 | 1 | 1 | 2 | 1 | 0 | 1 | 8 | low |
| Jara, et al., 2022c | 1 | 1 | 1 | 1 | 2 | 1 | 1 | 1 | 9 | low |
| Ma, et al., 2022 | 1 | 1 | 1 | 1 | 1 | 1 | 0 | 1 | 7 | low |
| Marra, et al., 2022 | 0 | 1 | 1 | 1 | 1 | 1 | 1 | 1 | 7 | low |
| Mirahmadizadeh, et al., 2022 | 1 | 1 | 1 | 1 | 0 | 1 | 1 | 1 | 7 | low |
| Nabirova, et al., 2022 | 1 | 1 | 1 | 1 | 0 | 1 | 1 | 1 | 7 | low |
| Paternina-Caicedo, et al., 2022 | 0 | 1 | 1 | 1 | 2 | 1 | 1 | 1 | 8 | low |
| Suah, et al., 2022 | 1 | 1 | 1 | 1 | 2 | 1 | 0 | 1 | 8 | low |
| Torres, et al., 2022 | 1 | 1 | 1 | 1 | 1 | 1 | 1 | 1 | 8 | low |
| Wu, et al., 2022 | 1 | 1 | 1 | 1 | 2 | 1 | 0 | 1 | 8 | low |
| Tak Cheng, et al., 2022 | 1 | 1 | 1 | 1 | 1 | 1 | 1 | 1 | 8 | low |
| Kang, et al., 2022 | 1 | 1 | 1 | 1 | 2 | 1 | 0 | 0 | 7 | low |

^a^A, Representativeness of the exposed cohort; B, Selection of the non-exposed cohort; C, Ascertainment of exposure; D, Demonstration that outcome of interest was not present at start of study.

^b^A, Comparability of cohorts on the basis of the design or analysis.

^c^A, Assessment of outcome; B, Was follow-up long enough for outcomes to occur; C, Adequacy of follow up of cohorts.

# Table S8. Quality assessment of case-control study

| **Study, Year (Reference)** | **Selection**^a^ | | | | **Comparability**^b^ | **Exposure**^c^ | | | **Total score** | **risk of bias** |
| --- | --- | --- | --- | --- | --- | --- | --- | --- | --- | --- |
|  | **A** | **B** | **C** | **D** | **A** | **A** | **B** | **C** |  |  |
| Hitchings, et al., 2021 | 1 | 1 | 1 | 0 | 2 | 1 | 1 | 1 | 8 | low |
| Ranzani, et al., 2021 | 1 | 1 | 1 | 1 | 1 | 1 | 1 | 1 | 8 | low |
| Belayachi, et al., 2022 | 1 | 0 | 1 | 1 | 2 | 1 | 1 | 1 | 8 | low |
| Cerqueira-Silva, et al., 2022a | 1 | 1 | 1 | 1 | 2 | 1 | 1 | 1 | 9 | low |
| Cerqueira-Silva, et al., 2022b | 1 | 1 | 1 | 0 | 2 | 1 | 1 | 1 | 8 | low |
| Cerqueira-Silva, et al., 2022c | 1 | 1 | 1 | 0 | 2 | 1 | 1 | 1 | 8 | low |
| Cerqueira-Silva, et al., 2022d | 1 | 1 | 1 | 0 | 2 | 1 | 1 | 1 | 8 | low |
| Institute of Public Health of Chile, 2022 | 1 | 1 | 0 | 1 | 2 | 1 | 1 | 1 | 8 | low |
| Mousa, et al., 2022 | 1 | 0 | 1 | 1 | 1 | 1 | 1 | 0 | 6 | moderate |
| Nadeem, et al., 2022 | 1 | 1 | 1 | 1 | 0 | 1 | 1 | 0 | 6 | moderate |
| Paixao, et al., 2022 | 1 | 0 | 1 | 1 | 2 | 1 | 1 | 1 | 8 | low |
| Ranzani, et al., 2022 | 1 | 1 | 1 | 1 | 2 | 1 | 1 | 1 | 9 | low |
| Rearte, et al., 2022 | 1 | 1 | 1 | 0 | 2 | 1 | 1 | 1 | 8 | low |
| Sritipsukho, et al., 2022 | 1 | 0 | 1 | 1 | 1 | 1 | 1 | 1 | 7 | low |
| Suphanchaimat, et al., 2022 | 1 | 1 | 1 | 1 | 1 | 1 | 1 | 1 | 8 | low |
| Suryatma, et al., 2022 | 1 | 1 | 1 | 1 | 1 | 1 | 1 | 1 | 8 | low |
| Zhang, et al., 2022 | 0 | 1 | 1 | 1 | 2 | 1 | 1 | 0 | 7 | low |
| Florentino, et al., 2022 | 1 | 0 | 1 | 1 | 2 | 1 | 1 | 1 | 8 | low |
| Yan, et al., 2022 | 1 | 1 | 1 | 1 | 1 | 1 | 1 | 1 | 8 | low |
| Wan, et al., 2022 | 1 | 0 | 1 | 1 | 1 | 1 | 1 | 1 | 7 | low |

^a^A, Is the case definition adequate?; B, Representativeness of the cases; C, Selection of Controls; D, Definition of Controls.

^b^A, Comparability of cases and controls on the basis of the design or analysis.

^c^A, Ascertainment of exposure; B, Same method of ascertainment for cases and controls; C, Non-Response rate.

# Table S9. Quality assessment of descriptive study

| **Study, Year (Reference)** | **A^a^** | **B** | **C** | **D** | **E** | **F** | **G** | **H** | **I** | **J** | **K** | **Total score** | **risk of bias** |
| --- | --- | --- | --- | --- | --- | --- | --- | --- | --- | --- | --- | --- | --- |
| Bayhan, et al., 2022 | 1 | 0 | 1 | 1 | 1 | 0 | 1 | 1 | 0 | 1 | 1 | 8 | low |
| Hu, et al., 2022 | 1 | 1 | 1 | 1 | 1 | 0 | 0 | 1 | 0 | 0 | 0 | 6 | moderate |
| McMenamin, et al., 2022 | 1 | 1 | 1 | 1 | 1 | 0 | 1 | 1 | 1 | 1 | 0 | 9 | low |
| Petrović, et al., 2022 | 1 | 1 | 1 | 1 | 1 | 0 | 1 | 1 | 0 | 1 | 1 | 9 | low |
| Toker, et al., 2022 | 1 | 1 | 1 | 1 | 1 | 1 | 1 | 0 | 0 | 1 | 0 | 8 | low |
| O, Zolt A, et al., 2022 | 1 | 1 | 1 | 1 | 1 | 0 | 1 | 1 | 0 | 0 | 1 | 8 | low |

^a^A, Define the source of information; B, List inclusion and exclusion criteria for exposed and unexposed subjects or refer to previous publications; C, Indicate time period used for identifying patients; D, Indicate whether or not subjects were consecutive if not population-based; E, Indicate if evaluators of subjective components of study were masked to other aspects of the status of the participants; F, Describe any assessments undertaken for quality assurance purposes; G, Explain any patient exclusions from analysis; H, Describe how confounding was assessed and/or controlled; I, If applicable, explain how missing data were handled in the analysis; J, Summarize patient response rates and completeness of data collection; K, Clarify what follow-up, if any, was expected and the percentage of patients for which incomplete data or follow-up was obtained.

# Table S10. Subgroup analysis of primary series VE against VOC

| **VOC** | **No.**  **estimates** | **Pooled estimate (95% CI)** | **VE% (95% CI)** | **I^2^ (%)** | **P value for heterogeneity** | **P value for subgroup differences** | **Weight (%)** |
| --- | --- | --- | --- | --- | --- | --- | --- |
| **Against SARS-CoV-2 Infection** | | | | | | | |
| Overall |  |  |  |  |  | <0.001 |  |
| Alpha | 2 | 0.280 (0.062, 1.273) | 72.0 (-27.3, 93.8) | 99.11 | <0.001 |  | 3.83 |
| Gamma | 7 | 0.558 (0.514, 0.606) | 44.2 (39.4, 48.6) | 73.95 | 0.001 |  | 12.81 |
| Delta | 26 | 0.472 (0.393, 0.567) | 52.8 (43.3, 60.7) | 99.64 | <0.001 |  | 43.27 |
| Omicron | 9 | 0.836 (0.772, 0.905) | 16.4 (9.5, 22.8) | 99.36 | <0.001 |  | 18.04 |
| Other | 12 | 0.422 (0.332, 0.535) | 57.8 (46.5, 66.8) | 99.65 | <0.001 |  | 22.05 |
| **Against Severe COVID-19** | | | | | | | |
| Overall |  |  |  |  |  | 0.295 |  |
| Alpha | 5 | 0.160 (0.059, 0.430) | 84.0 (57.0, 94.1) | 96.89 | <0.001 |  | 5.81 |
| Gamma | 7 | 0.267 (0.202, 0.353) | 73.3 (64.7, 79.8) | 97.21 | <0.001 |  | 11.13 |
| Delta | 31 | 0.306 (0.254, 0.369) | 69.4 (63.1, 74.6) | 94.25 | <0.001 |  | 37.96 |
| Omicron | 14 | 0.340 (0.299, 0.386) | 66.0 (61.4, 70.1) | 91.24 | <0.001 |  | 21.49 |
| Other | 15 | 0.351 (0.263, 0.469) | 64.9 (53.1, 73.7) | 99.14 | <0.001 |  | 23.61 |

# Table S11. Subgroup analysis of primary series VE by time since vaccination

| **Time since vac** | **No.**  **estimates** | **Pooled estimate**  **(95% CI)** | **VE (%) (95% CI)** | **I^2^ (%)** | **P value for heterogeneity** | **P value for subgroup differences** | **Weight (%)** |
| --- | --- | --- | --- | --- | --- | --- | --- |
| **Against** **SARS-CoV-2 Infection** | | | | | | | |
| Overall |  |  |  |  |  | <0.001 |  |
| 14-90 | 15 | 0.443 (0.356, 0.552) | 55.7 (44.8, 64.4) | 99.47 | <0.001 |  | 25.90 |
| 91-180 | 6 | 0.716 (0.561, 0.912) | 28.4 (8.8, 43.9) | 97.27 | <0.001 |  | 9.56 |
| >180 | 7 | 0.838 (0.731, 0.960) | 16.2 (4.0, 26.9) | 99.64 | <0.001 |  | 14.02 |
| Other | 28 | 0.439 (0.348, 0.555) | 56.1 (44.5, 65.2) | 99.88 | <0.001 |  | 50.52 |
| **Against Severe** **COVID-19** | | | | | | | |
| Overall |  |  |  |  |  | 0.021 |  |
| 14-90 | 16 | 0.222 (0.160, 0.307) | 77.8 (69.3, 84.0) | 97.85 | <0.001 |  | 20.64 |
| 91-180 | 8 | 0.390 (0.281, 0.541) | 61.0 (45.9, 71.9) | 97.32 | <0.001 |  | 10.53 |
| >180 | 7 | 0.403 (0.317, 0.512) | 59.7 (48.8, 68.3) | 98.72 | <0.001 |  | 12.01 |
| Other | 41 | 0.318 (0.278, 0.364) | 68.2 (63.6, 72.2) | 96.52 | <0.001 |  | 56.82 |

# **Table S****12. Subgroup analysis of booster VE against VOC**

| **VOC** | **No.**  **estimates** | **Pooled estimate**  **(95% CI)** | **VE (%) (95% CI)** | **I^2^ (%)** | **P value for heterogeneity** | **P value for subgroup differences** | **Weight (%)** |
| --- | --- | --- | --- | --- | --- | --- | --- |
| **Against SARS-CoV-2 Infection** | | | | | | | |
| Overall |  |  |  |  |  | <0.001 |  |
| Delta | 4 | 0.348 (0.234, 0.517) | 65.2 (48.3, 76.6) | 96.83 | <0.001 |  | 44.88 |
| Omicron | 5 | 0.797 (0.710, 0.895) | 20.3 (10.5, 28.0) | 93.05 | <0.001 |  | 55.12 |
| **Against Severe COVID-19** | | | | | | | |
| Overall |  |  |  |  |  | 0.130 |  |
| Delta | 4 | 0.208 (0.153, 0.283) | 79.2 (71.7, 84.7) | 73.95 | 0.009 |  | 33.01 |
| Omicron | 10 | 0.127 (0.073, 0.222) | 87.3 (77.8, 92.7) | 91.49 | <0.001 |  | 66.99 |

# Figure S1. Funnel plot for VE against SARS-CoV-2 infection

**
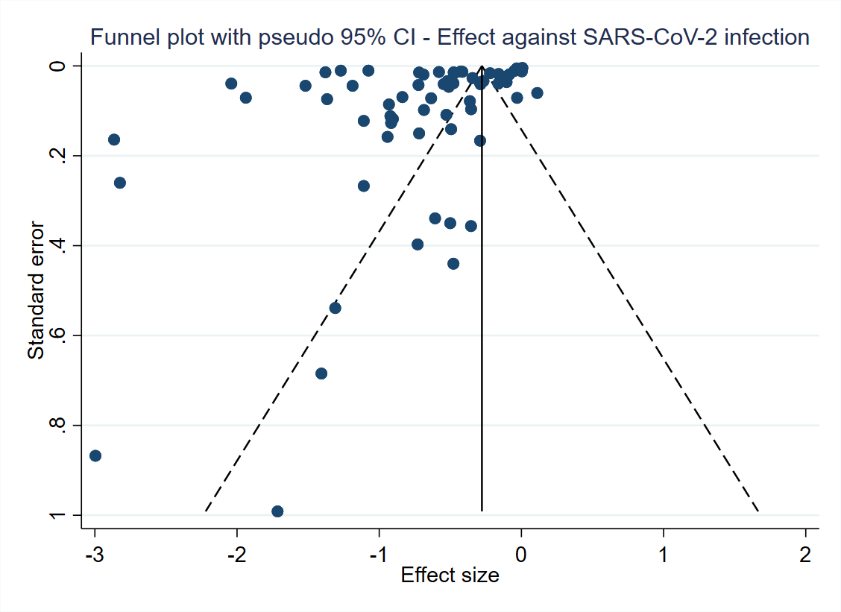
**

# Figure S2. Funnel plot for VE against severe COVID-19

**
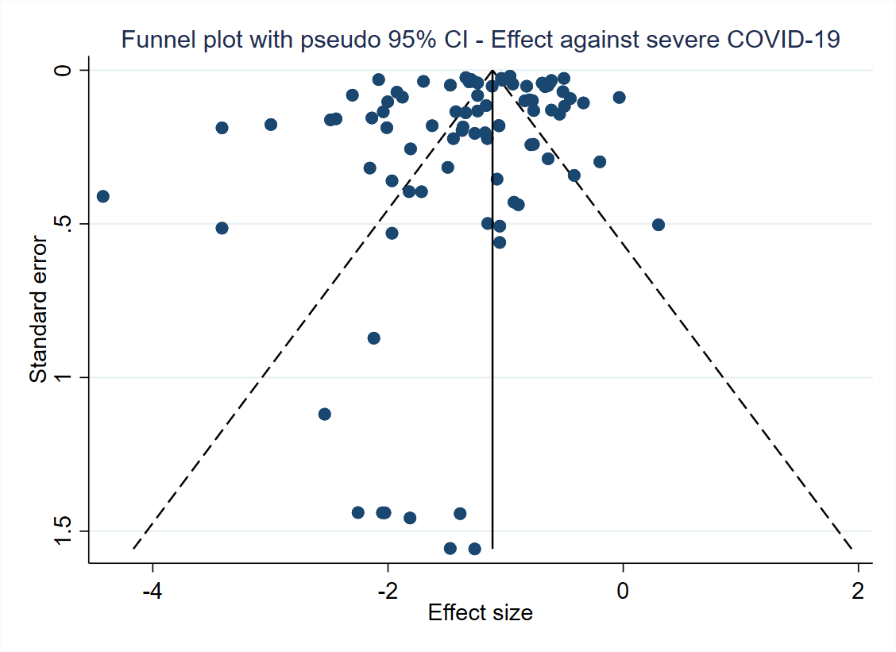
**

# Table S13. Sensitivity analysis (deleting moderate risk of bias data): meta-regression of VE against SARS-CoV-2 infection

| **Factors** | **b** | **95% CI** | **P** | **S.E.** |
| --- | --- | --- | --- | --- |
| **Study region** | | | | |
| Western Pacific Region | ref | - | - | - |
| Region of Americas | -0.084 | (-0.484, 0.316) | 0.675 | 0.199 |
| European Region | -0.025 | (-0.484, 0.433) | 0.913 | 0.229 |
| Region of South-East Asia | -0.059 | (-0.647, 0.528) | 0.841 | 0.293 |
| Eastern Mediterranean Region | -0.385 | (-1.064, 0.294) | 0.260 | 0.338 |
| **VOC** | | | | |
| Alpha | ref | - | - | - |
| Gamma | 0.720 | (-0.177, 1.617) | 0.113 | 0.447 |
| Delta | 0.437 | (-0.331, 1.206) | 0.259 | 0.383 |
| Omicron | 1.052 | (0.203, 1.901) | 0.016 | 0.423 |
| Other | 0.432 | (-0.412, 1.277) | 0.309 | 0.421 |
| **Time since vaccination** | | | | |
| 14-90 | ref | - | - | - |
| 91-180 | 0.532 | (0.000, 1.063) | 0.050 | 0.532 |
| >180 | 0.560 | (0.110, 1.010) | 0.016 | 0.560 |
| Other | 0.194 | (-0.133, 0.521) | 0.240 | 0.194 |
| **Constant** | -1.368 | (-2.191, -0.545) | 0.002 | -1.368 |

This model had △AICc value=10.817 and R^2^=29.62% among all potential models. Model with the lowest AICc value included factors “VOC” and “Time since vaccination”.

# Table S14. Sensitivity analysis (deleting moderate risk of bias data): meta-regression of VE against severe COVID-19

| **Factors** | **b** | **95% CI** | **P** | **S.E.** |
| --- | --- | --- | --- | --- |
| **Study region** | | | | |
| Western Pacific Region | ref | - | - | - |
| Region of Americas | 0.391 | (0.023, 0.805) | 0.064 | 0.207 |
| European Region | 2.518 | (1.710, 3.327) | <0.001 | 0.404 |
| Region of South-East Asia | -0.065 | (-0.783, 0.653) | 0.857 | 0.359 |
| Eastern Mediterranean Region | 0.077 | (-0.426, 0.580) | 0.760 | 0.252 |
| **VOC** | | | | |
| Alpha | ref | - | - | - |
| Gamma | 0.812 | (0.114, 1.509) | 0.023 | 0.349 |
| Delta | 1.067 | (0.509, 1.624) | <0.001 | 0.279 |
| Omicron | 1.144 | (0.507, 1.780) | <0.001 | 0.318 |
| Other | 0.459 | (-0.220, 1.138) | 0.181 | 0.340 |
| **Population type** | | | | |
| general | ref | - | - | - |
| HCWs | 0.573 | (-0.890, 2.035) | 0.437 | 0.731 |
| Covid19 inpatient | 1.291 | (0.705, 1.877) | <0.001 | 0.293 |
| chronical patient | 0.445 | (-0.190, 1.081) | 0.166 | 0.318 |
| **Age range** | | | | |
| 18-59 | ref | - | - | - |
| <18 | -0.531 | (-1.147, 0.086) | 0.090 | 0.308 |
| ≥60 | 0.541 | (0.267, 0.814) | <0.001 | 0.137 |
| Other | 0.518 | (-0.138, 1.173) | 0.120 | 0.328 |
| **Vaccine doses** | | | | |
| primary | ref | - | - | - |
| booster | -0.633 | (-0.978, -0.288) | <0.001 | 0.173 |
| **Study design** | | | | |
| cohort study | ref | - | - | - |
| retrospective cohort study | 0.863 | (0.441, 1.285) | <0.001 | 0.211 |
| case-control study | 0.144 | (-0.236, 0.524) | 0.453 | 0.190 |
| descriptive study | -1.861 | (-2.661, -1.061) | <0.001 | 0.400 |
| **Constant** | -2.766 | (-3.505, -2.028) | <0.001 | 0.369 |

This model had △AICc value=0 and the highest R^2^=60.45% among top potential best models.

# Table S15. Sensitivity analysis (deleting outliers): meta-regression of VE against SARS-CoV-2 infection

| **Factors** | **b** | **95% CI** | **P** | **S.E.** |
| --- | --- | --- | --- | --- |
| **Study region** | | | | |
| Western Pacific Region | ref | - | - | - |
| Region of Americas | -0.127 | (-0.544, 0.291) | 0.546 | 0.208 |
| European Region | 0.015 | (-0.463, 0.493) | 0.949 | 0.238 |
| Region of South-East Asia | -0.037 | (-0.651, 0.577) | 0.904 | 0.306 |
| Eastern Mediterranean Region | -0.812 | (-1.445, -0.178) | 0.013 | 0.316 |
| **VOC** | | | | |
| Alpha | ref | - | - | - |
| Gamma | 0.182 | (-0.351, 0.716) | 0.496 | 0.266 |
| Delta | -0.160 | (-0.557, 0.237) | 0.423 | 0.198 |
| Omicron | 0.518 | (0.083, 0.954) | 0.021 | 0.217 |
| **Time since vaccination** | | | | |
| 14-90 | ref | - | - | - |
| 91-180 | 0.481 | (-0.078, 1.039) | 0.090 | 0.278 |
| >180 | 0.525 | (0.051, 0.998) | 0.031 | 0.236 |
| Other | 0.074 | (-0.277, 0.426) | 0.673 | 0.175 |
| **Constant** | -0.744 | (-1.287, -0.202) | 0.008 | 0.270 |

This model had △AICc value=3.835 and the highest R^2^=34.00% among top potential best models.

# Table S16. Sensitivity analysis (deleting outliers): meta-regression of VE against severe COVID-19

| **Factors** | **b** | **95% CI** | **P** | **S.E.** |
| --- | --- | --- | --- | --- |
| **Study region** | | | | |
| Western Pacific Region | ref | - | - | - |
| Region of Americas | 0.331 | (0.019, 0.643) | 0.038 | 0.156 |
| European Region | 2.674 | (2.036, 3.313) | <0.001 | 0.319 |
| Region of South-East Asia | -0.033 | (-0.566, 0.501) | 0.903 | 0.266 |
| Eastern Mediterranean Region | -0.091 | (-0.455, 0.274) | 0.620 | 0.182 |
| **VOC** | | | | |
| Alpha | ref | - | - | - |
| Gamma | 1.153 | (0.402, 1.905) | 0.003 | 0.375 |
| Delta | 1.390 | (0.720, 2.059) | <0.001 | 0.335 |
| Omicron | 1.603 | (0.874, 2.333) | <0.001 | 0.365 |
| Other | 0.682 | (-0.057, 1.422) | 0.070 | 0.369 |
| **Population type** | | | | |
| general | ref | - | - | - |
| HCWs | 0.525 | (-0.731, 1.781) | 0.406 | 0.627 |
| Covid19 inpatient | 1.291 | (0.830, 1.753) | <0.001 | 0.230 |
| chronical patient | 0.359 | (-0.201, 0.918) | 0.204 | 0.279 |
| **Age range** | | | | |
| 18-59 | ref | - | - | - |
| <18 | 0.122 | (-0.906, 1.149) | 0.813 | 0.513 |
| ≥60 | 0.477 | (0.274, 0.681) | <0.001 | 0.101 |
| Other | 0.758 | (-0.478, 1.993) | 0.224 | 0.617 |
| **Vaccine doses** | | | | |
| primary | ref | - | - | - |
| booster | -0.709 | (-0.984, -0.435) | <0.001 | 0.137 |
| **Study design** | | | | |
| cohort study | ref | - | - | - |
| retrospective cohort study | 0.734 | (0.386, 1.083) | <0.001 | 0.174 |
| case-control study | -0.074 | (-0.377, 0.229) | 0.628 | 0.151 |
| descriptive study | -2.072 | (-2.709, -1.435) | <0.001 | 0.318 |
| **Constant** | -2.874 | (-3.680, -2.068) | <0.001 | 0.403 |

This model had △AICc value=0 and the highest R^2^=77.51% among top potential best models.
